# *Gardnerella vaginalis*, *Fannyhessea vaginae*, and *Prevotella bivia* Strongly Influence Each Other's Transcriptome in Triple-Species Biofilms

**DOI:** 10.1007/s00248-024-02433-9

**Published:** 2024-09-19

**Authors:** Lúcia G. V. Sousa, Juliano Novak, Angela França, Christina A. Muzny, Nuno Cerca

**Affiliations:** 1 Centre of Biological Engineering (CEB), Laboratory of Research in Biofilms Rosário Oliveira (LIBRO), University of Minho, Braga, Portugal; 2 Botucatu Medical School, Department of Pathology, São Paulo State University (UNESP), Botucatu, SP Brazil; 3 LABBELS – Associate Laboratory, Braga, Portugal; 4 Division of Infectious Diseases, University of Alabama at Birmingham, Birmingham, AL USA

**Keywords:** Bacterial vaginosis, Polymicrobial biofilms, RNA-sequencing, Species interactions

## Abstract

Bacterial vaginosis (BV), the most common vaginal infection worldwide, is characterized by the development of a polymicrobial biofilm on the vaginal epithelium. While *Gardnerella* spp. have been shown to have a prominent role in BV, little is known regarding how other species can influence BV development. Thus, we aimed to study the transcriptome of *Gardnerella vaginalis*, *Fannyhessea vaginae*, and *Prevotella bivia*, when growing in triple-species biofilms. Single and triple-species biofilms were formed in vitro, and RNA was extracted and sent for sequencing. cDNA libraries were prepared and sequenced. Quantitative PCR analysis (qPCR) was performed on the triple-species biofilms to evaluate the biofilm composition. The qPCR results revealed that the triple-species biofilms were mainly composed by *G. vaginalis* and *P. bivia* was the species with the lowest percentage. The RNA-sequencing analysis revealed a total of 432, 126, and 39 differentially expressed genes for *G. vaginalis*, *F. vaginae*, and *P. bivia*, respectively, when growing together. Gene ontology enrichment of *G. vaginalis* downregulated genes revealed several functions associated with metabolism, indicating a low metabolic activity of *G. vaginalis* when growing in polymicrobial biofilms. This work highlighted that the presence of 3 different BV-associated bacteria in the biofilm influenced each other’s transcriptome and provided insight into the molecular mechanisms that enhanced the virulence potential of polymicrobial consortia. These findings will contribute to understand the development of incident BV and the interactions occurring within the biofilm.

**Supplementary Information:**

The online version contains supplementary material available at 10.1007/s00248-024-02433-9.

## Introduction

Bacterial vaginosis (BV) is the most common vaginal infection worldwide with a prevalence of approximately 30% [1, 2]. BV is associated with multiple adverse gynecologic and obstetrical outcomes, including pelvic inflammatory disease [3], preterm birth [4], infertility [5], an increased risk of HIV [6], and other sexually transmitted infections [7–9]. BV presents a high economic burden mainly due to lack of treatment success and high rates of recurrence [10]. Despite multiple decades of research on BV pathogenesis, the exact etiology of this infection remains unknown. More than two hundred different bacterial species have been identified in the vaginal microbiota of women with BV [11, 12], including several strict and facultative anaerobic bacteria which replace protective lactic acid–producing *Lactobacillus* species that colonize the vagina [13]. Among these diverse bacterial species, key BV-associated bacteria (BVAB) have been found in the majority of the BV cases and may be important in the development of infection, such as *Gardnerella*, *Fannyhessea* (previously known as *Atopobium* [14]), *Prevotella*, *Mobiluncus*, *Peptostreptococcus*, *Megasphaera*, *Sneathia*, *Candidatus* Lachnocurva vaginae (previously known as BVAB 1 [15]), *Amygdalobacter indicium* (previously known as BVAB 2 [16]), and *Mageeibacillus indolicus* (previously known as BVAB 3 [17]) [18, 19]. The development of a polymicrobial biofilm in the vaginal epithelium of women with BV is the most noteworthy characteristic of this infection [20], and has been associated with the lack of treatment success and high recurrence rates after treatment [21, 22]. Thus, the BV biofilm is an important virulence factor that requires further investigation [23]. Studying key BVAB present in the BV biofilm is vital to better understanding the mechanisms of BV development and identifying potential virulence factors that could be the target of new treatment options [24].

*Gardnerella* spp., present in 95–100% of cases of BV [25–27], have been the focus of previous transcriptomic studies showing important virulence traits in this species’ transcriptome [28]. Our prior study found that the expression of some genes involved in antimicrobial resistance, biofilm formation, epithelial adhesion, and evasion of the immune response were upregulated in *Gardnerella* biofilms comparing to planktonic cells, suggesting that this phenotype may contribute towards the chronic and recurrent nature of BV [28].

To our knowledge, no studies have evaluated the transcriptome of multiple key BVAB. A previous study suggested that *G. vaginalis*, *P. bivia*, and *F. vaginae* may have an essential role in the initial development of incident BV (iBV) [29]. Thus, the major goal of this work was to study the transcriptome of these three key BVAB, namely *G. vaginalis*, *F. vaginae*, and *P. bivia*, when cultured in single-species versus triple-species biofilms, and identify genes important for virulence.

## Materials and Methods

### Bacterial Growth Conditions

*G. vaginalis* ATCC 14018, *F. vaginae* ATCC BAA-55, and *P. bivia* ATCC 29303 were stored in brain heart infusion medium (Liofilchem, Roseto degli Abruzzi, Italy) supplemented with 23% of glycerol (98%, Panreac, Darmstadt, Germany) at − 80 °C. They were grown on Columbia base agar (Liofilchem) plates with 5% of defibrinated horse blood (CBA) (Thermo Fisher Scientific, Lenexa, KS) for 48 h at 37 °C and in anaerobic conditions (Anaerocult™ A, Merck Millipore, Taufkirchen, Germany).

### Biofilm Formation

Single- and triple-species biofilms of *G. vaginalis*, *F. vaginae*, and *P. bivia* were formed on 24-well culture plates (Orange Scientific, Braine-l’Alleud, Belgium) using the competitive model previously described [30]. First, an inoculum of each species was prepared in NYCIII medium and incubated at 37 °C in anaerobic conditions for 24 h. Thereafter, the bacterial concentration was adjusted to 9 × 10^7^ CFU/mL by reading the optical density at 620 nm [31]. The bacterial suspensions were then dispensed on the plate wells for a total volume of 1 mL and a final concentration of 1 × 10^7^ CFU/mL, and incubated for 48 h at 37 °C and in anaerobic conditions. For quantitative polymerase chain reaction (qPCR) experiments, the medium was removed and the biofilms were washed once with 0.9% NaCl (Sigma, Germany). Then, the biofilms were mechanically detached from the plates in 1 mL of NaCl and the content of the wells combined. For RNA-seq experiments, the medium was removed, and the biofilms were washed once with 1 × phosphate-buffered saline (PBS) solution (Thermo Fisher Scientific). Following the washing steps RNA Protect bacteria reagent (Qiagen, MD, USA), diluted 2:1 in PBS, was dispensed on the biofilms and the biofilms were detached from the plate. The samples were then centrifuged at 5000 g for 10 min at room temperature. These experiments were repeated at least three times.

### Determination of Biofilm Composition by qPCR

Triple-species biofilms prepared as described above were used for quantification of each species by qPCR. The concentration of each species in the triple-species biofilm was determined using the calibration curves previously designed using the same approach for genomic DNA (gDNA) extraction and quantification by qPCR [32]. Briefly, after detaching the biofilms from the plate, 900 μL of the triple-species biofilm suspension was added to a new tube containing 100 μL of *Escherichia coli* ATCC 25992 suspension (for a final concentration of 1 × 10^8^ CFU/mL). GDNA was extracted from triple-species biofilms using the DNeasy UltraClean microbial Kit (Qiagen) following the manufacturer’s instructions with minor optimizations. After extraction, gDNA was diluted 40 × for qPCR experiments. The primers used to quantify *G. vaginalis*, *F. vaginae*, *P. bivia* [33], and *E. coli* [34] have been previously described. The qPCR experiments were performed on a CFX Connect Real-Time PCR Detection System (Bio-Rad, CA, USA) with the following cycle parameters: 95 °C for 3 min, and 40 cycles of 95 °C for 5 s and 60 °C for 20 s. The cycle threshold (CT) obtained for each species, at each time point, was normalized as a relative quantification to the exogenous control *E. coli*, using the formula E^ΔCT^, where E stands for the reaction efficiency and ΔCT = (CT_target_ – CT _exogenous control_).

### RNA Extraction

Twelve biofilms of each condition were pooled to obtain samples with enough RNA concentration for further analysis. RNA extraction was performed using the RNeasy Mini Kit (Qiagen), according to the manufacturer’s instructions, as optimized before [35]. First, cells were suspended in 600 μL of lysis buffer RLT, and the volume was transferred to a tube with 0.1-mm zirconium beads (Merck, Darmstadt, Germany). Cells were lysed using the BeadBug 6 Microtube Homogenizer (Benchmark Scientific, NJ, USA) at maximum speed for 35 s. The cycle was repeated four times and the samples kept on ice for 5 min between cycles. Then, the samples were centrifuged, and the supernatant recovered into a new tube. Ethanol at 70% (Thermo Fisher Scientific) was added in the same proportion (vol:vol) to the supernatant, and the solution was transferred to an RNeasy Mini spin column. After the washing steps in the column, the RNA was eluted in RNase-free water (Grisp). RNA was treated with Turbo DNase (Invitrogen, Waltham, MA, USA) to degrade genomic DNA following the manufacturer’s instructions for rigorous protocol.

### cDNA Library Preparation and Sequencing

RNA quality was assessed using the Agilent 2100 Bioanalyzer (Agilent, CA, USA) and only samples with RNA quality indicators above 7 were used. RNA-seq libraries were prepared using Lexogen’s CORALL™ Total RNA-seq kit (Lexogen, Vienna, Austria) with 100 ng of total RNA from each sample. Before RNA-seq, rRNA was removed using the RiboCop for Bacteria (mixed bacterial samples META) rRNA Depletion kit (Lexogen). Sequencing libraries were evaluated for quality on a Fragment Analyzer System (Agilent) and quantified with Qubit™ dsDNA HS Assay Kit (Invitrogen).

Sequencing data were generated using Illumina NextSeq 2000 Sequencing from single-end reads (SR100). FastQ files were generated via Illumina bcl2fastq2 (v.2.17.1.14). The quality of individual sequences was evaluated using FastQC software after adapter trimming with cutadapt software (1.18).

### RNA-Sequencing Data Analysis

FastQ files were then analyzed using CLC Genomics Workbench software (Qiagen, version 21.99). Quality trimming, including both quality scores and nucleotide ambiguity, was performed using the CLC genomics workbench default settings (Supplementary Table 1). Alignment of each species’ sequences was performed using *G. vaginalis* NCTC10287 (NCBI reference sequence: NZ_LR134385.1), *F. vaginae* FDAARGOS_934 (NCBI reference sequence: CP065631.1), and *P. bivia* DSM 20514 (NCBI reference sequence: NZ_JH660658.1; NZ_JH660659.1; NZ_JH660660.1) as reference genomes, also using default settings (Supplementary Table 2). Differential expression analysis was performed using reads per kilobase per million (RPKM)–mapped fragments as the normalization strategy using the single-species biofilms as controls. Baggerley’s test was applied to identify statistically significant alterations. Fold changes > 2 or <  − 2 and with a false discovery rate (FDR) *p* value < 0.05 were considered significant and used for further bioinformatics analyses. Raw and analyzed datasets were deposited in the Gene Expression Omnibus database under the reference GSE268115.

### Functional Annotation

Functional enrichment of differentially expressed genes (DEGs) was assessed using the Search Tool for the Retrieval of Interacting Genes/Proteins (STRING, version 11.5) based on Gene Ontology (GO) and Kyoto Encyclopedia of Genes and Genomes (KEGG) databases. Classes with FDR-adjusted *p* value < 0.05 were considered for enrichment. REVIGO was used for removing redundant GO terms. UniProt was used to find the homology of hypothetical proteins.

### RNA-seq Data Validation by qPCR

Several DEGs from the three species were chosen based on their function and levels of differential expression. The genes were then used for validation of the RNA sequencing data, using the same RNA used for sequencing (technical validation) and RNA obtained from other independent experiments (biological validation). Primers for the selected genes were designed with the Primer3 tool (version 4.1.0) and are described in Supplementary Information. RNA was reverse transcribed using the Xpert cDNA Synthesis Kit (Grisp) for 400 ng of total RNA. qPCR was then performed for quantification of the expression of the selected genes using 2 μL of cDNA samples (diluted 1:100) and 8 μL of qPCR mix containing 5 μL of Xpert fast SYBR mix (Grisp), 1 μL of primers, and 2 μL of DNase/RNase-free water. No-template controls were included to evaluate reagent contamination. No-reverse control was used to assess contamination with gDNA. The conditions used for qPCR runs were the same as described above on qPCR experiments for biofilm quantification. Melt analysis was performed to ensure the absence of unspecific products and primer dimers. Gene expression was determined by the relative quantification method using the formula E^ΔCT^, where E stands for the reaction efficiency and ΔCT = (CT_target gene_ – CT_reference gene (16S rRNA)_).

### Statistical Analysis

The principal component analysis (PCA) graphs and heatmaps were created using the CLC Genomics Workbench (version 21.99). Gene interaction networks were constructed using Cytoscape software (version 3.10.0) with the STRING app, and major networks were obtained with the MCODE app. All the other figures and analyses were performed using GraphPad Prism version 8.2 (La Jolla, CA, USA). Statistical analysis was performed using one-way ANOVA with Tukey’s multiple comparisons test. Statistical differences were considered when *p* < 0.05.

## Results

### Biofilm Composition

**Fig. 1:**
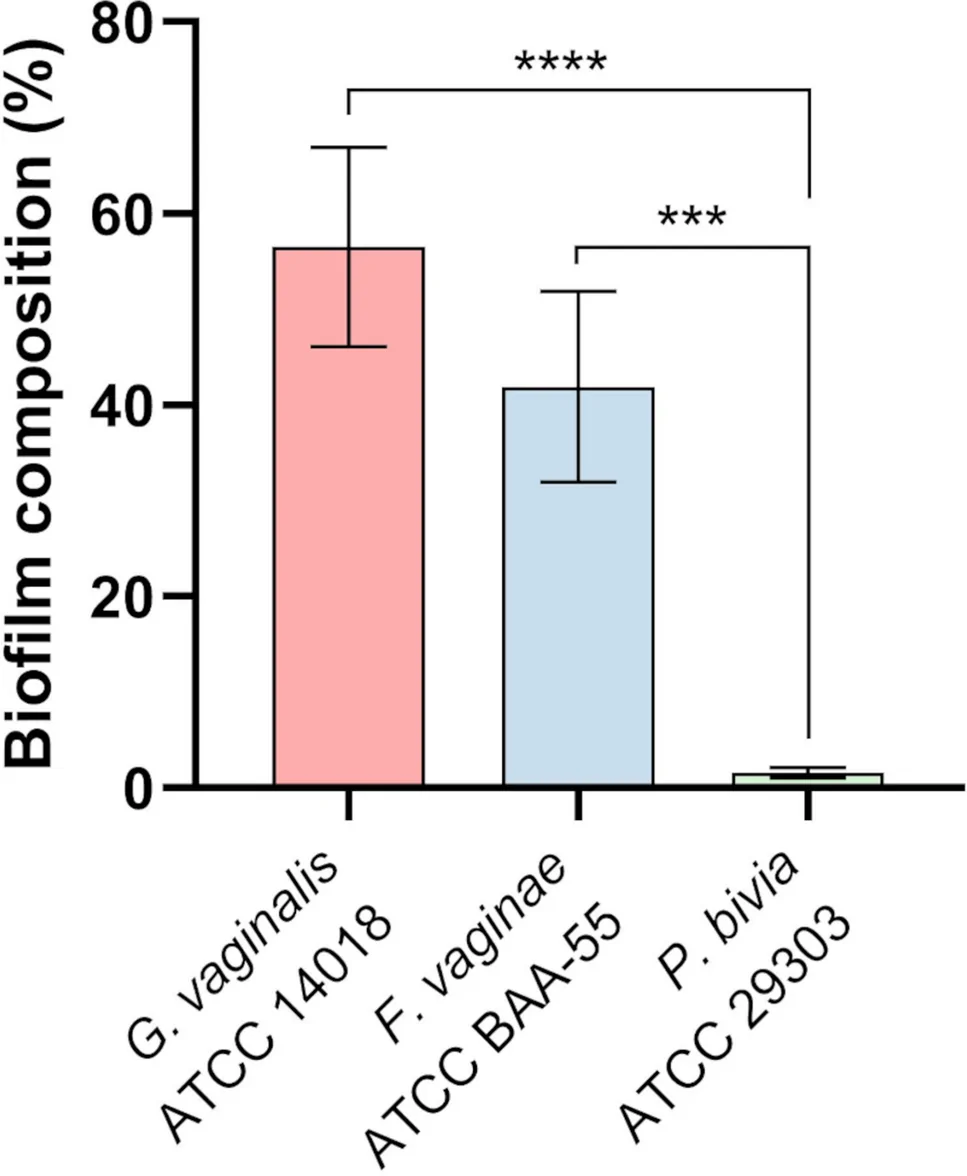
Triple-species biofilms composition determined by qPCR. Statistical analysis was performed using the one-way ANOVA. Differences between each species composition are represented by * (*** *p* < 0.001; **** *p* < 0.0001). The graphic was plotted using GraphPad Prism

The biofilms composition, determined by qPCR (Fig. 1), demonstrated that 57% of the polymicrobial biofilm was composed of *G. vaginalis*, followed by 42% of *F. vaginae*, while only 2% was composed of *P. bivia*.

### General Properties of the Transcriptome

The quality of the sequencing data was trimmed, producing an average of 15,945,719, 17,008,832, 17,745,831, and 17,730,992 reads for *G. vaginalis*, *F. vaginae*, *P. bivia*, and the triple-species consortium, respectively (Supplementary Table 3). The trimmed reads were then mapped to a reference genome of *G. vaginalis*, *F. vaginae*, and *P. bivia* (Table 1). The percentage of reads mapped was highly variable ranging from 1.82% (for *P. bivia* on triple-species conditions on scaffold 2) to 88.34% (for *G. vaginalis* on single-species conditions). *F. vaginae* sequences had the highest percentage of reads mapped to the reference genome on both single- and triple-species biofilm conditions. In general, single-species biofilm libraries had higher percentages of mapping than triple-species libraries.

**Table 1:** Read mapping summary statistics for *Gardnerella vaginalis*, *Fannyhessea vaginae*, and *Prevotella bivia* on single-species and triple-species biofilms. The results represent the average for the three analyzed replicates

		Reads mapped			Reads not mapped			Total		
Species	Condition	Average Nr sequences	SD	%	Average Nr sequences	SD	%	Average Nr sequences	SD	%
*G. vaginalis*	Single	14,086,012.67	785,569.86	88.34	1,859,706.33	70,349.01	11.66	15,945,719.00	850,082.41	100.00
	Triple	1,738,180.33	944,937.31	9.80	15,992,809.33	2,816,433.46	90.20	17,730,989.67	3,760,521.62	100.00
*F. vaginae*	Single	14,979,010.67	2,247,051.64	88.07	2,029,821.00	367,164.16	11.93	17,008,831.67	2,474,223.45	100.00
	Triple	13,433,645.33	2,080,592.37	75.76	4,297,344.33	1,680,029.00	24.24	17,730,989.67	3,760,521.62	100.00
*P. bivia* (scaffold 1)^a^	Single	6,790,239.33	631,865.77	38.26	10,955,591.00	2,063,761.14	61.74	17,745,830.33	2,066,402.73	100.00
	Triple	1,017,553.67	212,958.04	5.74	16,713,436.00	3,565,808.03	94.26	17,730,989.67	3,760,521.62	100.00
*P. bivia* (scaffold 2)^a^	Single	2,330,204.67	875,981.97	13.13	15,415,625.67	1,323,200.45	86.87	17,745,830.33	2,066,402.73	100.00
	Triple	322,683.67	76,964.77	1.82	17,408,306.00	3,683,717.48	98.18	17,730,989.67	3,760,521.62	100.00
*P. bivia* (scaffold 3)^a^	Single	12,909,967.33	948,777.86	72.75	4,835,863.00	1,249,455.05	27.25	17,745,830.33	2,066,402.73	100.00
	Triple	2,034,453.67	487,665.46	11.47	15,696,536.00	3,283,018.80	88.53	17,730,989.67	3,760,521.62	100.00

^a^*P. bivia* mapping sequence is divided into 3 scaffolds

PCA plots revealed a high correlation of gene expression within the three single-species replicates analyzed for *G. vaginalis* (Supplementary Fig. 1), and *F. vaginae* (Supplementary Fig. 2). In contrast, on the triple-species, two replicates were closely related while one showed more differences. For *P. bivia* (Supplementary Fig. 3–5), the three scaffolds showed different distributions, and, in general, the single-species triplicates had more variation, indicating poor consistency between replicates.

**Fig. 2:**
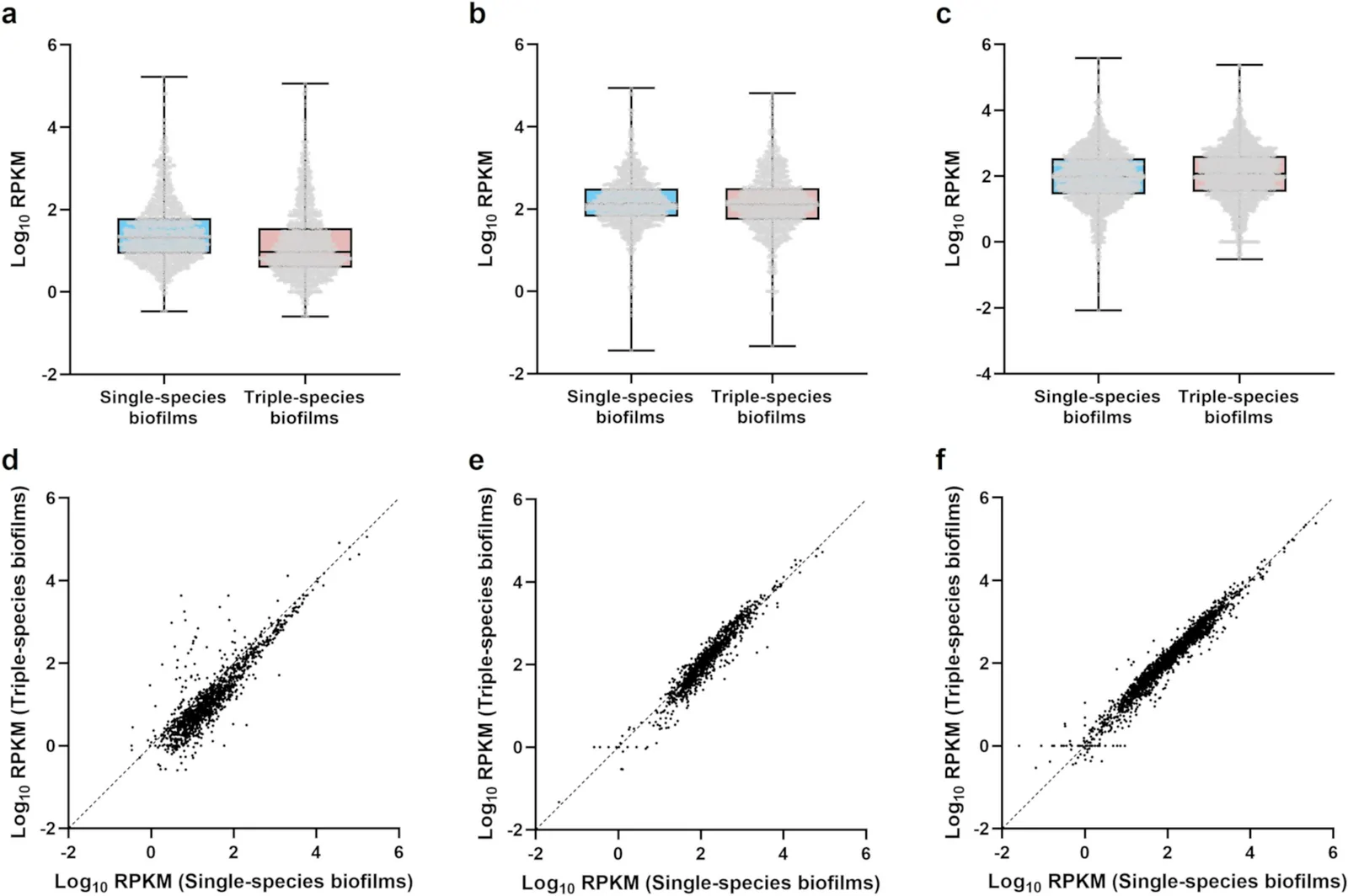
Density distribution of reads per kilobase per million (RPKM) values among conditions. Distribution of RPKM values on the single-species and triple-species biofilms for *Gardnerella vaginalis* (**a**), *Fannyhessea vaginae* (**b**), and *Prevotella bivia* (**c**). Correlation between RPKM values on the single- and triple-species biofilms for *G. vaginalis* (**d**), *F. vaginae* (**e**), and *P. bivia* (**f**). Graphics were plotted using GraphPad Prism

The density distribution and correlation of RPKM between single- and triple-species conditions can be observed in Fig. 2. While the gene distribution showed consistency among the two conditions for the three species (Fig. 2a, b, and c), the correlation of expression levels between the single-species and triple-species conditions varied (Fig. 2d, e, and f). In the cases of *F. vaginae* and *P. bivia*, genes had similar values of expression on the single and triple-species conditions since the majority of the genes displayed along the curve. However, for *G. vaginalis* more genes had distinct expression levels on the two conditions, as can be seen by the spreading of the values around the curve.

### Analysis of Differentially Expressed Genes

**Fig. 3:**
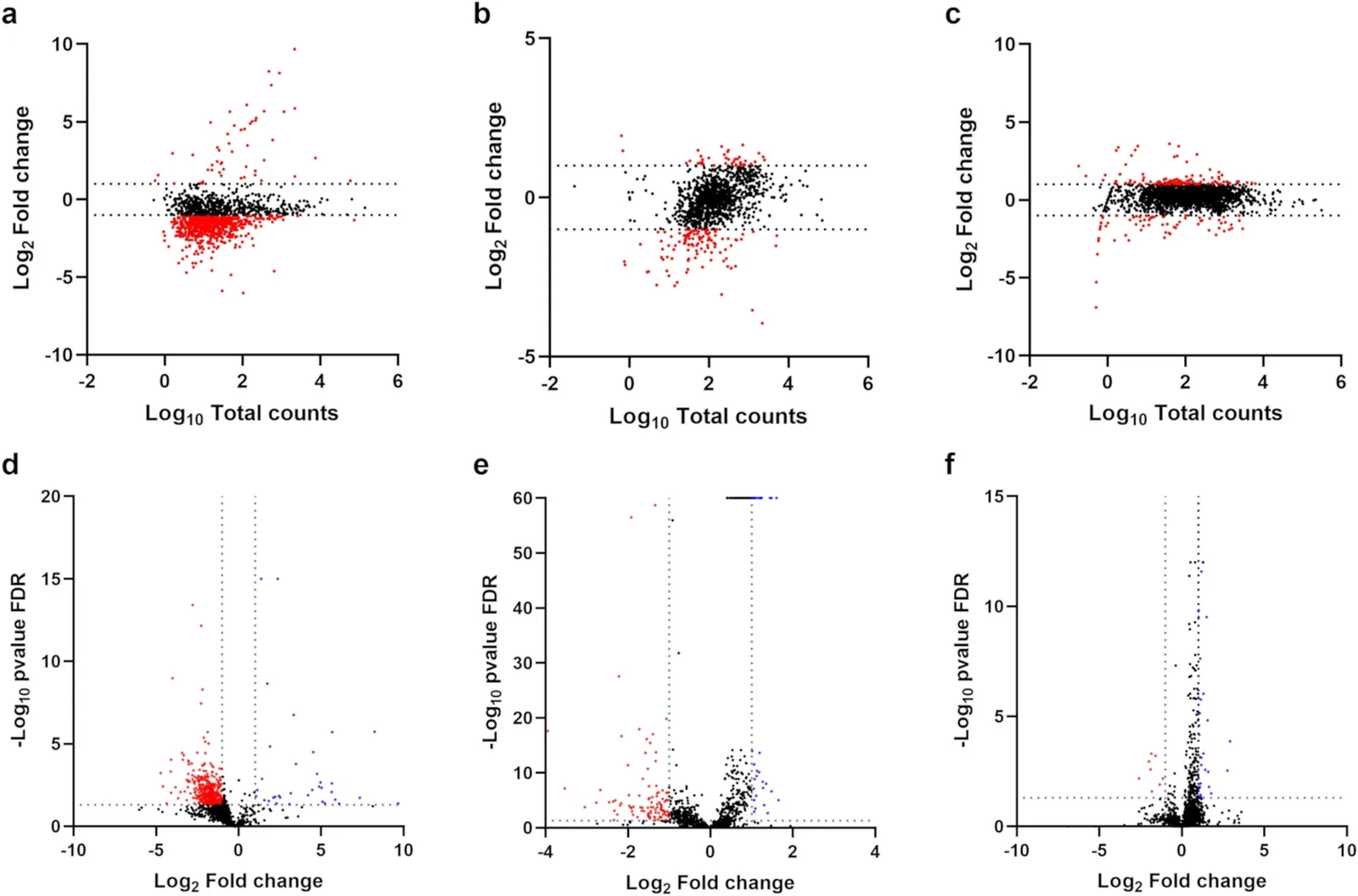
Analysis of differentially expressed genes. Distribution of differentially expressed genes by MA plots for *Gardnerella vaginalis* (**a**), *Fannyhessea vaginae* (**b**), and *Prevotella bivia* (**c**) and by volcano plots for *G. vaginalis* (**d**), *F. vaginae* (**e**), and *P. bivia* (**f**). Graphics were plotted using GraphPad Prism

The MA and volcano plots in Fig. 3 revealed the organization of the DEGs for the three species using the single-species biofilms as a control. A total of 1315, 1202, and 2184 genes were identified for *G. vaginalis*, *F. vaginae*, and *P. bivia*, respectively, after mapping to the reference genomes. For *G. vaginalis*, more genes were downregulated on the triple-species condition. For *F. vaginae* and *P. bivia*, less DEGs were identified, and while *F. vaginae* had more downregulated genes, *P. bivia* showed more upregulated genes.

The heatmaps and clustering trees also revealed the differences in the gene expression of each species when growing on single-species or triple-species biofilms. For *G. vaginalis* (Supplementary Fig. 6), the single-species triplicates were closely related and showed similar gene expression, while the triple-species replicates had more variability. The *F. vaginae* single- and triple-species replicates were very homogeneous (Supplementary Fig. 7). For *P. bivia* (Supplementary Fig. 8–10), it was possible to observe that two of the single-species replicates (S2 and S3) were very similar, as well as the triple-species replicates (M2 and M3). The other two replicates (S1 and M1), showed similarities however were more distant from the respective replicates.

**Fig. 4:**
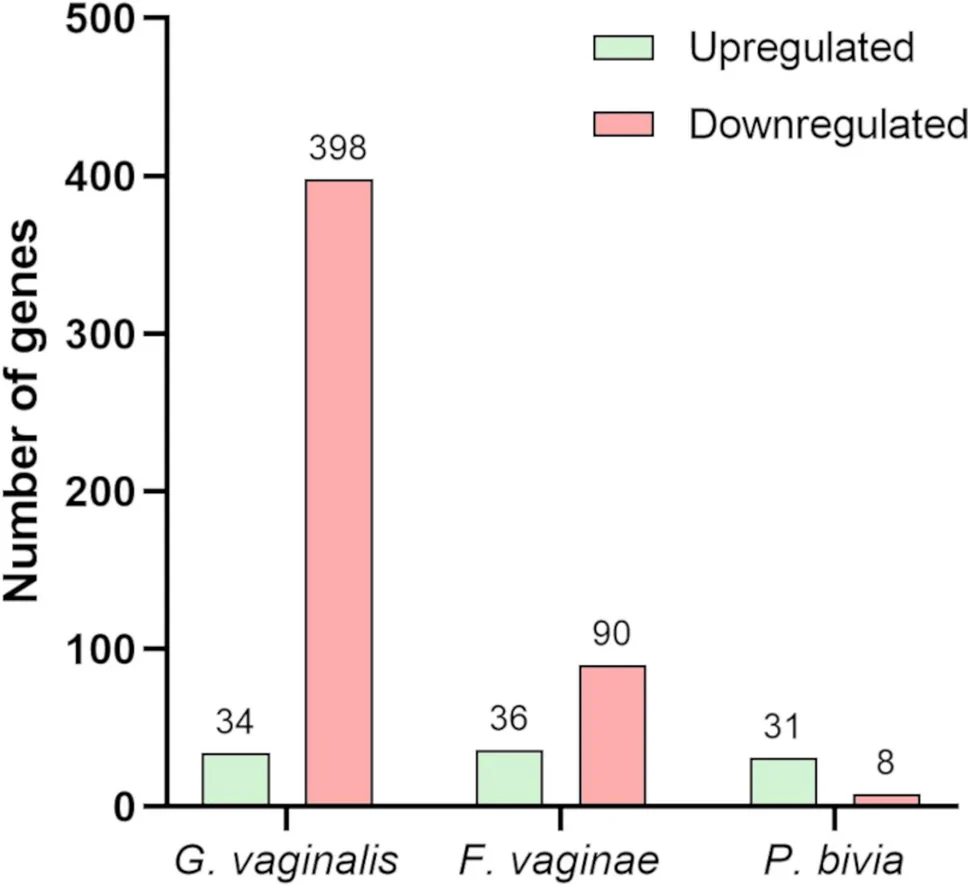
Number of upregulated and downregulated genes for each species on triple-species biofilms. Significant differential gene expression was considered for values of fold change > 2 or <  − 2 and with FDR *p* value < 0.05. The graphic was plotted using GraphPad Prism

Genes with values of fold change > 2 or < − 2 and FDR *p* value < 0.05 were considered as significantly differentially expressed and used for further analysis. The number of significantly DEGs in each of the species is represented in Fig. 4. A total of 432, 126, and 39 DEGs were observed for *G. vaginalis*, *F. vaginae*, and *P. bivia*, respectively. The top 10 most upregulated and downregulated genes for each species are represented in Table 2.

**Table 2:** List of 10 most upregulated and downregulated genes in *Gardnerella vaginalis*, *Fannyhessea vaginae*, and *Prevotella bivia*

Gene	Description	Fold change	FDR *p* value
***G. vaginalis*–upregulated genes**			
EL180_RS06735	Prevent-host-death protein	815.08	4.08 × 10^−2^
EL180_RS07255	Hypothetical protein	302.58	1.86 × 10^−6^
EL180_RS06515	Amino acid ABC transporter ATP-binding protein	162.75	1.85 × 10^−2^
EL180_RS05445	ATP-binding protein	67.43	4.25 × 10^−2^
EL180_RS05315	Type II toxin-antitoxin system RelB/DinJ family antitoxin	58.12	2.42 × 10^−2^
EL180_RS05410	GNAT family N-acetyltransferase	51.12	1.96 × 10^−6^
EL180_RS05440	Type II toxin-antitoxin system RelB/DinJ family antitoxin	50.19	2.52 × 10^−3^
EL180_RS05320	Type II toxin-antitoxin system RelE/ParE family toxin	49.87	8.51 × 10^−3^
EL180_RS05310	CadD family cadmium resistance transporter	37.51	4.01 × 10^−2^
EL180_RS06745	CadD family cadmium resistance transporter	33.92	3.23 × 10^−2^
***G. vaginalis*–downregulated genes**			
EL180_RS06590	DUF881 domain–containing protein	− 26.36	6.03 × 10^−4^
EL180_RS07325	Hypothetical protein	− 23.89	3.92 × 10^−3^
EL180_RS02430	Hypothetical protein	− 20.48	4.07 × 10^−2^
EL180_RS06320	DUF3046 domain–containing protein	− 17.26	4.75 × 10^−4^
EL180_RS01485	Acyltransferase	− 17.08	9.05 × 10^−5^
EL180_RS00850	DUF4125 family protein	− 17.00	9.35 × 10^−3^
EL180_RS06595	CDP-alcohol phosphatidyltransferase family protein	− 16.04	1.06 × 10^−9^
EL180_RS03360	CrcB family protein	− 14.14	4.24 × 10^−3^
EL180_RS00940	YggT family protein	− 12.71	1.99 × 10^−2^
* rimM*	Ribosome maturation factor RimM	− 11.49	8.45 × 10^−3^
***F. vaginae*–upregulated genes**			
I6G91_04240	HlyC/CorC family transporter	3.14	9.68 × 10^−6^
I6G91_01720	Metal ABC transporter permease	3.04	0.00
I6G91_01715	ATP-binding cassette domain–containing protein	2.80	0.00
I6G91_01120	PTS sugar transporter subunit IIA	2.80	2.45 × 10^−7^
I6G91_01725	Hypothetical protein	2.71	0.00
* rpoD*	RNA polymerase sigma factor RpoD	2.62	2.11 × 10^−3^
I6G91_00155	Hypothetical protein	2.56	1.02 × 10^−8^
I6G91_01130	PTS sugar transporter subunit IIB	2.46	7.72 × 10^−5^
* argF*	Ornithine carbamoyltransferase	2.43	5.63 × 10^−8^
I6G91_02330	Acetate kinase	2.42	4.38 × 10^−9^
***F. vaginae*–downregulated genes**			
I6G91_05895	LytTR family transcriptional regulator	− 15.41	2.41 × 10^−18^
I6G91_05900	DUF3021 domain–containing protein	− 11.60	6.96 × 10^−8^
I6G91_05165	LacI family DNA–binding transcriptional regulator	− 8.29	1.73 × 10^−4^
I6G91_04355	Plasmid mobilization relaxosome protein MobC	− 5.13	1.61 × 10^−5^
I6G91_04350	Relaxase/mobilization nuclease domain–containing protein	− 4.80	3.98 × 10^−3^
* rbsK*	Ribokinase	− 4.65	2.71 × 10^−28^
I6G91_04445	ABC transporter ATP–binding protein	− 4.56	8.53 × 10^−5^
I6G91_05905	Hypothetical protein	− 4.51	3.69 × 10^−5^
I6G91_04380	Phosphate ABC transporter substrate–binding protein	− 4.47	2.02 × 10^−17^
I6G91_05185	DeoR/GlpR transcriptional regulator	− 4.00	4.10 × 10^−12^
***P. bivia*–upregulated genes**			
* pdxT*	Pyridoxal 5'-phosphate synthase glutaminase subunit PdxT	3.43	3.14 × 10^−2^
PREBIDRAFT_RS06525	Cob(I)yrinic acid a,c-diamide adenosyltransferase	3.14	1.59 × 10^−2^
* pdxS*	Pyridoxal 5'-phosphate synthase lyase subunit PdxS	3.03	3.29 × 10^−3^
PREBIDRAFT_RS05230	Inorganic phosphate transporter	2.94	1.52 × 10^−5^
PREBIDRAFT_RS01305	YaaA family protein	2.83	3.13 × 10^−10^
PREBIDRAFT_RS05120	Bifunctional hydroxymethylpyrimidine kinase/phosphomethylpyrimidine kinase	2.68	2.70 × 10^−3^
PREBIDRAFT_RS05580	Glycosyltransferase family 87 protein	2.49	4.81 × 10^−4^
PREBIDRAFT_RS05685	C1 family peptidase	2.48	9.43 × 10^−7^
PREBIDRAFT_RS05235	DUF47 family protein	2.45	0.00
PREBIDRAFT_RS11735	Hypothetical protein	2.41	0.00
***P. bivia*–downregulated genes**			
PREBIDRAFT_RS00795	Hypothetical protein	− 6.02	6.61 × 10^−3^
PREBIDRAFT_RS05900	RNA polymerase sigma factor	− 4.01	1.11 × 10^−3^
PREBIDRAFT_RS05895	Hypothetical protein	− 3.74	2.56 × 10^−3^
PREBIDRAFT_RS05910	DUF4252 domain–containing protein	− 3.61	4.86 × 10^−4^
PREBIDRAFT_RS05890	DUF4252 domain–containing protein	− 3.52	2.54 × 10^−2^
PREBIDRAFT_RS05905	DUF4252 domain–containing protein	− 3.04	6.13 × 10^−4^
PREBIDRAFT_RS05630	Peptidase U32 family protein	− 2.53	1.25 × 10^−2^
PREBIDRAFT_RS06070	Spore maturation protein	− 2.22	6.74 × 10^−3^

### Enrichment of Differentially Expressed Genes

**Fig. 5:**
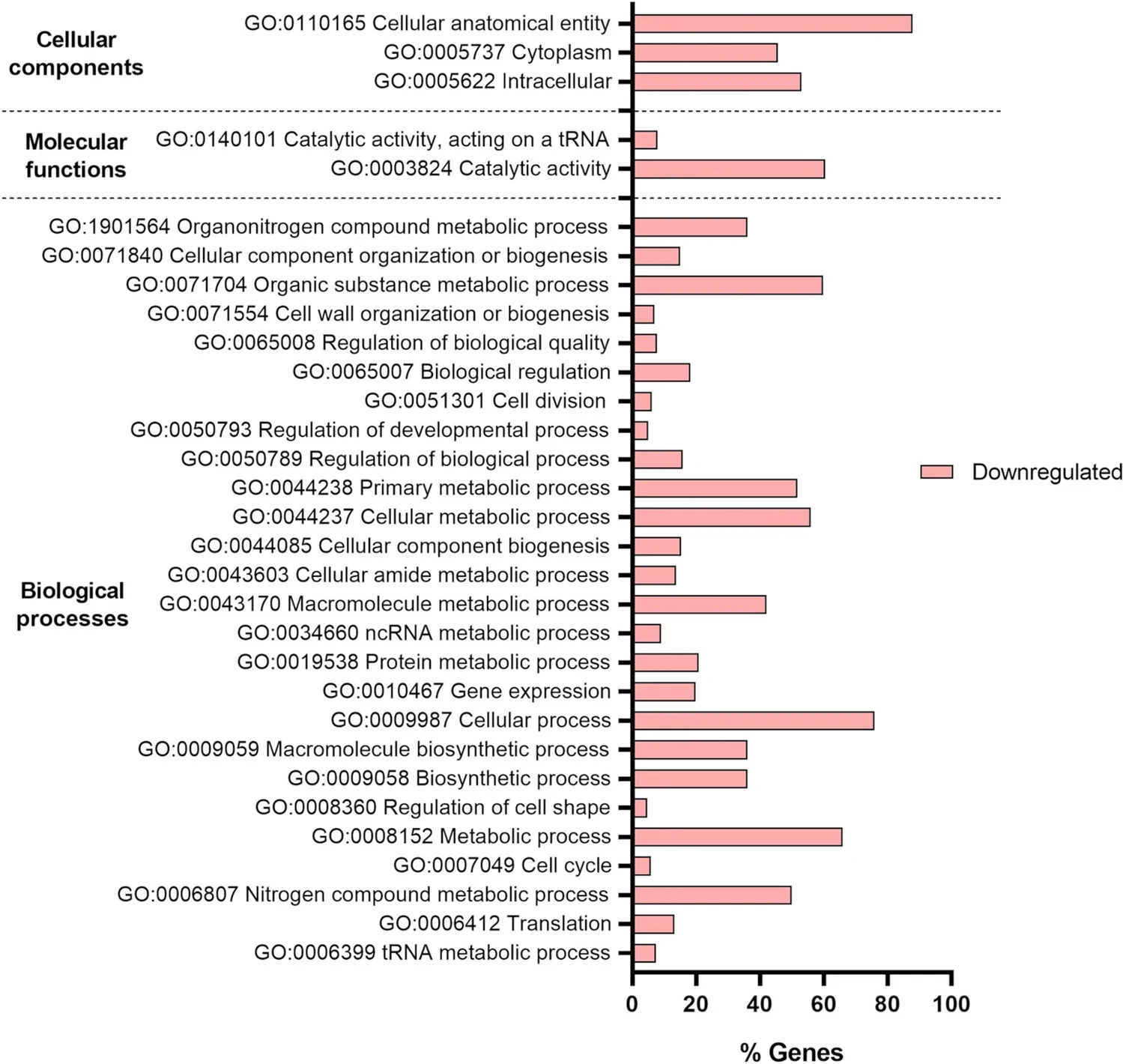
Gene ontology  analysis for *Gardnerella vaginalis* downregulated genes. The figure was created using GraphPad Prism

A GO analysis of the DEGs revealed that only the *G. vaginalis* genes were enriched and, curiously, enrichment was only found for the downregulated genes, with 47 GO terms identified. Two terms were associated with molecular functions, 3 with cellular components, and 42 with biological processes. A REVIGO analysis eliminated 16 redundant terms, with the cured GO analysis represented in Fig. 5. Terms associated with biological processes, mainly metabolism, were downregulated in triple-species biofilms when compared with single-species biofilms, suggesting that *G. vaginalis* cells are less metabolically active in the triple-species biofilms. The main clusters in the upregulated and downregulated genes in *G. vaginalis*, *F. vaginae*, and *P. bivia* are shown in Supplementary Fig. 11.

### Validation of RNA-seq Data by qPCR

**Fig. 6:**
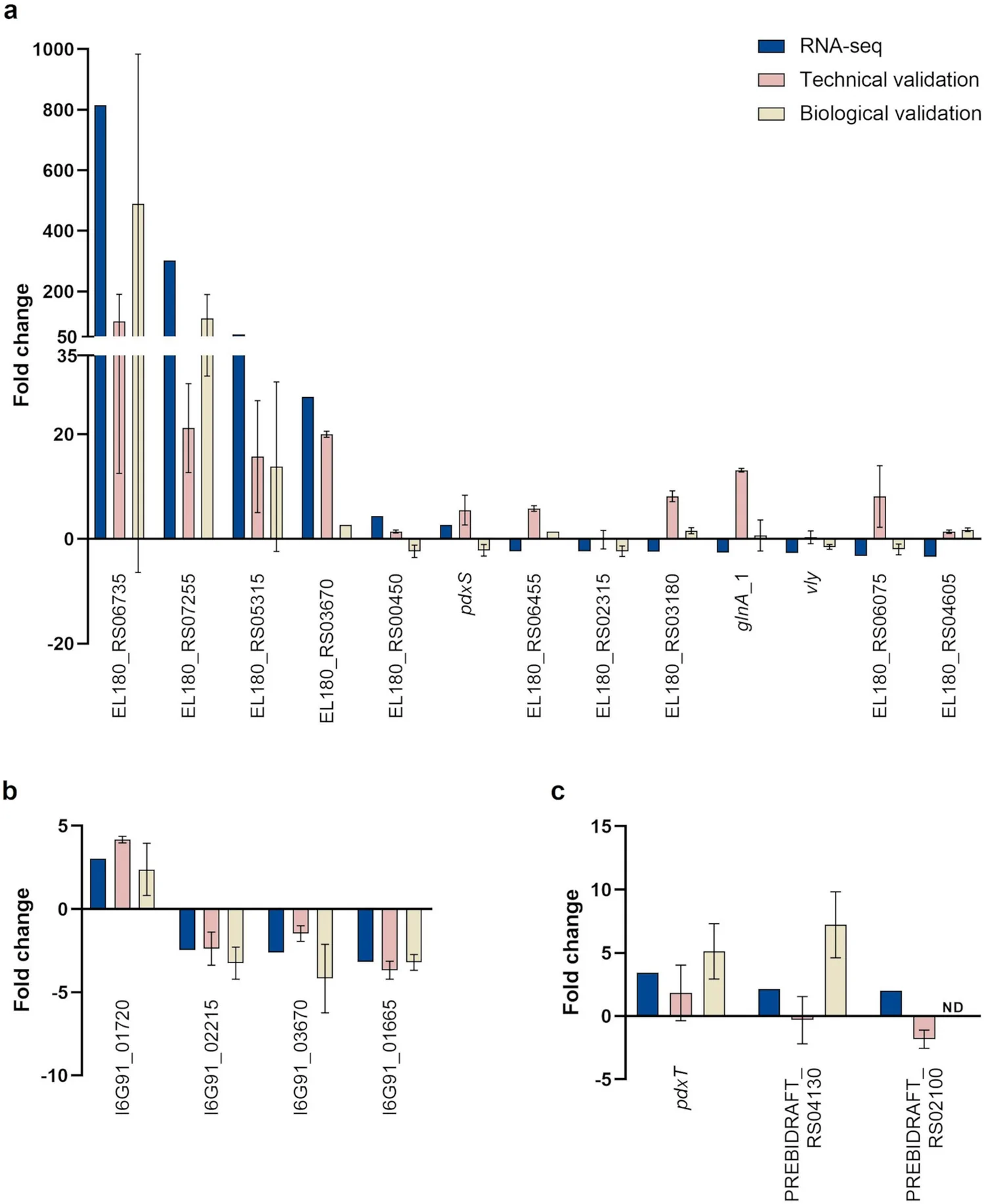
Fold change of gene expression (triple-species vs. single-species biofilms) determined by RNA-seq and qPCR. The validation was performed by qPCR on the same samples used for RNA-seq (technical validation) and on other samples extracted in the same conditions (biological replicates). The results represent fold change for *Gardnerella vaginalis* (**a**), *Fannyhessea vaginae* (**b**), and *Prevotella bivia* (**c**). ND: non-detected. Graphics were plotted using GraphPad Prism

To confirm the RNA-seq data by qPCR, we selected different genes, as described in Supplementary Table 4. Looking for potential molecular markers for the diagnosis of BV, we first analyzed the 5 most upregulated genes from *G. vaginalis*. We also included other DEGs associated with important biological functions such as membrane transport, adhesion, and biofilm formation. The list of primers for the genes of interest is described in Supplementary Table 5. Unfortunately, we were not able to successfully optimize primers for all the genes of interest, as described in the Supplementary Methods. First, to ensure that the results obtained by RNA-seq were accurate (technical validation), we determined the expression of the selected genes by qPCR using the same RNA utilized to construct RNA-seq libraries (Supplementary Table 6). As can be observed, some of the genes were not detected by qPCR, or when a product was formed, the melting curve analysis revealed more than 1 amplicon, which prevented us from accurately assessing the fold change values on these samples. Thus, a total of 13 *G. vaginalis*, 4 *F. vaginae*, and 3 *P. bivia* genes were successfully used in the qPCR validation. Second, to confirm that the results obtained by RNA-seq were reproducible (biological validation), the fold change expression of the selected genes was determined using new RNA samples extracted under identical conditions (the results for both validations are shown on Fig. 6). We noted that, while the four highest DEGs of *G. vaginalis* demonstrated a consistent trend between RNA-seq and qPCR, for the remaining selected genes, in certain instances of technical or biological validation, the fold change values exhibited an opposite trend*.* In the case of *F. vaginae*, the fold change alterations detected by qPCR were similar to the ones obtained by RNA-seq. However, for *P. bivia*, the 3 selected genes presented 3 different profiles: while the expression of the *pdxT* gene showed the same trend in all 3 conditions, the technical validation of the PREBIDRAFT_RS04130 gene revealed non-differential expression (despite the same upregulation trend being observed in both RNA-seq and biological validation). The PREBIDRAFT_RS02100 gene was not detected in the biological replicates while simultaneously presenting an opposite trend to the RNA-seq data on the technical validation.

## Discussion

To understand how *G. vaginalis*, *F. vaginae*, and *P. bivia* interact when growing on polymicrobial biofilms, we analyzed the transcriptome of single- and triple-species biofilms. Differential gene expression analysis used the single-species biofilms as a control, to assess how a specific species is influenced when growing in the presence of other species. As expected, *G. vaginalis* was dominant in the triple-species biofilms [30, 31].

The transcriptomic analysis revealed that, in some situations, the percentage of transcripts mapped was low. Furthermore, variability was observed in the triplicates of some conditions, as shown on the PCA plots, as well as on the gene expression between triplicates, as represented on the heatmaps.

A smaller number of statistically DEGs were identified for *F. vaginae* and *P. bivia*, compared to the number of genes found for *G. vaginalis*. For *G. vaginalis* and *F. vaginae*, the majority of DEGs was downregulated, while in the case of *P. bivia* was upregulated.

*G. vaginalis* was the only species where enrichment was detected, only among the downregulated genes; these genes were mainly related to metabolic functions. It was previously shown that *G. vaginalis* biofilm cells present a lower metabolic activity when compared to the planktonic mode of growth [28], but this was not surprising, since many other species have also shown a similar trend, such as *Helicobacter pylori* [36] or *Porphyromonas gingivalis* [37]. Nevertheless, here, we have shown that the metabolic activity of *G. vaginalis* biofilm cells was lower when in the presence of the other two species in the biofilm. A similar result was obtained for *Staphylococcus aureus* when cultured with *S. epidermidis* in dual-species biofilms; *S. aureus* showed a down-regulation of genes associated with metabolism when in dual-species biofilms [38].

Taking into consideration the pivotal role of *G. vaginalis* in BV-associated biofilms, we analyzed the expression of some *G. vaginalis* virulence genes, namely vaginolysin (*vly*) and sialidase. In the case of *vly*, a cholesterol-dependent cytolysin that has a role in lysis of vaginal epithelial cells [39], it was found to be downregulated in the triple-species biofilms compared to the single-species biofilms of *G. vaginalis*. Our previous study analyzing the expression of *vly* in dual- and triple-species biofilms reported no differences in *vly* expression in dual-species biofilms formed by *G. vaginalis* and *F. vaginae* or *P. bivia*, compared to *G. vaginalis* single-species biofilms. However, a slight downregulation was observed in triple-species biofilms formed by these three BVAB [31].

The gene encoding for sialidase, an enzyme known for the destruction of the protective mucus layer on the vaginal epithelium [40], was found as non-statistically significant (FDR *p* value > 0.05) on our experimental setup. Previously, we observed a slight downregulation of the gene encoding for sialidase in a triple-species biofilm formed by *G. vaginalis*, *F. vaginae*, and *P. bivia* [31]. However, in our earlier work, we used a pre-conditioned *G. vaginalis* biofilm model, wherein a *G. vaginalis* single-species biofilm was first formed, and 24 h later *F. vaginae* and *P. bivia* were introduced. Despite triple-species biofilms formed in either model resulting in similar bacterial species composition, the biogeography of each model is strikingly different [30]. Taking into consideration that different types of interactions among bacterial species not only result in different spatial organization of the species within biofilms [41, 42] but also influence gene expression [43–45], the differences between these two studies are not surprising.

Although most of the highlighted genes in this study belonged to *G. vaginalis*, both *F. vaginae* and *P. bivia* were also affected by the presence of the other species. For *F. vaginae*, among the top 10 most upregulated and downregulated genes, several associated with membrane transport (including I6G91_04240 and I6G91_01720) were upregulated on the triple-species biofilms while several associated with transcriptional regulation were downregulated (namely I6G91_05895, I6G91_05165, and I6G91_05185). Despite not having any enrichment detected for the DEGs of *F. vaginae*, the analysis on Cytoscape allowed the identification of the main clusters for the upregulated and downregulated genes. The main cluster on the upregulated genes was associated with the PTS system transporter and membrane biogenesis protein, whereas for the downregulated genes the main cluster was associated with phosphate ABC transporter and two-component sensor histidine kinase.

*P. bivia* was the species with the least number of DEGs detected, and, although no similar functions were found within the 10 most upregulated and downregulated genes, we noted on the analysis of the main clusters the identification of the upregulated genes, *atpA*, *rpsL*, and *tuf*, and the identification of the downregulated genes associated with the DUF4252 domain-containing protein and RNA polymerase sigma factor. However, for these two species, little is known about important genes that might play a role in bacterial virulence; thus, the identification of genes of interest for these species was limited.

Since *Gardnerella* species are commonly found in BV and exhibit an important role in biofilm formation, we sought to identify potential molecular markers for diagnosing BV, focusing on highly upregulated genes from *G. vaginalis*. Despite the initial selection of the top five candidates, due to the limitations on primers optimization (as described in the Supplementary Methods), it was only possible to quantify by qPCR three of the five most upregulated genes as potential molecular diagnostic markers (EL180_RS06735, EL180_RS07255, and EL180_RS05315). Notably, these 3 genes displayed high values of fold change in both RNA-seq and qPCR analyses. Other genes, namely EL180_RS06075 and EL180_RS04605, associated with membrane transport and antibiotic resistance, did not show consistency between RNA-seq and qPCR (in both technical and biological validations). The gene EL180_RS03670, which is also related to membrane transport, was upregulated on the triple-species biofilms, and the orthologous gene was also upregulated on biofilm cells in the previous RNA-seq work from our group [28]. Curiously, a gene associated with biofilm formation, EL180_RS06455, that was upregulated in biofilms when compared to planktonic cells [28], was found downregulated in the triple-species biofilms, suggesting that the other two species present on the biofilm might influence gene expression. Another gene selected for qPCR validation was EL180_RS00450, which encodes for an internalin protein. This gene was found to be associated with host cell invasion in *Listeria monocytogenes* [46]. We performed an analysis of protein homology using UniProt which revealed that the protein from *G. vaginalis* shares an identity of 27–58.3% with different internalin proteins from *L. monocytogenes* (data not shown). Thus, this protein might play a similar role in *G. vaginalis*. However, this gene was consistently upregulated across all replicates during technical validation but showed downregulation in the biological validation samples.

We also verified that the qPCR validation on the technical replicates was not always concordant with the RNA-seq results. A previous bioinformatics study comparing expression data generated by wet-lab validated qPCR of 18,080 protein-coding genes by RNA-seq and qPCR, showed that 15–20% of the analyzed genes had opposite differential gene expression between the two techniques [47]. Furthermore, we observed that the values of fold change in the biological validation did not show the same tendency as the fold change for the technical replicates in this study. However, in this case, the biological variability inherent to the biofilm formation experiments may have the main influence on the differences observed.

Overall, this work highlights the adaptation of 3 key BVAB when growing in a triple-species biofilm. The transcriptomic analysis led to the identification of several DEGs that need further investigation, particularly in in vivo studies, to evaluate their impact on BV progression and biofilm formation.

## Supplementary Information

Below is the link to the electronic supplementary material.

Supplementary file1 (DOCX 2.35 MB)

## Data Availability

The RNA-seq datasets generated and/or analysed during this study are available in the Gene Expression Omnibus repository, under the reference GSE268115. Further datasets used and/or analyzed during this study are available from the corresponding author upon reasonable written request.
