# Supplementary material for: Gardnerella vaginalis, Fannyhessea vaginae, and Prevotella bivia Strongly Influence Each Other's Transcriptome in Triple-Species Biofilms
Source: Microb Ecol. 2024 Sep 19;87(1):117. doi: 10.1007/s00248-024-02433-9 (PMC11410844; doi:10.1007/s00248-024-02433-9)
Supplement: Supplementary file 1 — Supplementary file1 (DOCX 2.35 MB) [file 248_2024_2433_MOESM1_ESM.docx]

***Gardnerella vaginalis*, *Fannyhessea vaginae*, and *Prevotella bivia* Strongly Influence Each Other’s Transcriptome In Triple-Species Biofilms**

**Lúcia G. V. Sousa^1^, Juliano Novak^1,2^, Angela França^1,3^, Christina A. Muzny^4^, Nuno Cerca^1,3#^**

^1^Centre of Biological Engineering (CEB), Laboratory of Research in Biofilms Rosário Oliveira (LIBRO), University of Minho, Braga, Portugal.

^2^São Paulo State University (UNESP), Botucatu Medical School, Department of Pathology, Botucatu, SP, Brazil.

^3^LABBELS – Associate Laboratory, Braga, Portugal.

^4^Division of Infectious Diseases, University of Alabama at Birmingham, Birmingham, AL, USA.

# **#Corresponding Author**

Nuno Cerca, PhD

Email address: nunocerca@ceb.uminho.pt

**Supplementary materials and methods**

**Primer design and optimization**

Primers for 16 *Gardnerella vaginalis* genes, 4 *Fannyhessea vaginae* genes, and 3 *Prevotella bivia* genes were designed using Primer 3 tool. Due to the high diversity of species that can be associated with bacterial vaginosis, the specificity of the primers was only theoretically assessed using the BLAST tool from NCBI.

The efficiency of the primers was first evaluated using cDNA prepared from RNA from single-species biofilms of each of the species, with serial dilutions of 1/5. However, for some of the primers we observed that they could not be optimized in these conditions, either because the primers did not quantify the gene in the samples or because they presented different melting temperatures, meaning that different products were amplified. For those primers, a new set of primers was designed using a different region of the gene sequence or increasing the number of base pairs. The determination of efficiency was repeated using cDNA prepared from RNA from single-species biofilms. Although some of the primers could be optimized, others still presented the same problems as described before. For those cases, a new set of primers was prepared and the efficiency was determined. For the specific cases of primers that could not be optimized in these conditions, the cDNA was prepared using gene-specific primers (GSP), which allowed it to surpass some of the previous limitations and the remaining primers could be optimized to obtain satisfactory values of efficiency. At this time, we had some primers that were optimized using cDNA prepared with random primers and others using cDNA prepared with GSP. Supplementary Table 5 presents the list of primers used to target each of the selected genes and if they were optimized using cDNA with random primers or GSP.

| **Quality trimming** | |
| --- | --- |
| Quality limit (Phred scale) | 0.05 |
| **Trim ambiguous nucleotides** | |
| Maximum number of ambiguities allowed | 2 |

**Supplementary Table 1** Trimming parameters applied for the analysis of RNA-seq raw reads using CLC Genomics Workbench (version 21.99)

| **Condition** | **Replicates** | **Nr of reads** | **Avg. Length** | **Nr of reads after trim** | **Percentage trimmed** | **Avg. Length after trim** |
| --- | --- | --- | --- | --- | --- | --- |
| *G. vaginalis* | S1 | 15,296,536 | 89 | 15,296,536 | 100 | 88.80 |
|  | S2 | 15,632,689 | 89 | 15,632,689 | 100 | 88.10 |
|  | S3 | 16,907,933 | 89 | 16,907,932 | 100 | 88.78 |
| *F. vaginae* | S1 | 14,191,738 | 89 | 14,191,738 | 100 | 88.76 |
|  | S2 | 18,829,409 | 89 | 18,829,409 | 100 | 88.81 |
|  | S3 | 18,005,348 | 89 | 18,005,348 | 100 | 88.80 |
| *P. bivia* | S1 | 19,269,918 | 89 | 19,269,917 | 100 | 88.79 |
|  | S2 | 18,573,721 | 89 | 18,573,721 | 100 | 88.77 |
|  | S3 | 15,393,853 | 89 | 15,393,853 | 100 | 88.81 |
| Triple-species | M1 | 13,507,629 | 89 | 13,507,626 | 100 | 88.82 |
|  | M2 | 18,968,645 | 89 | 18,968,645 | 100 | 88.82 |
|  | M3 | 20,716,702 | 89 | 20,716,698 | 100 | 88.80 |

**Supplementary Table 2** Mapping parameters applied for the analysis of RNA-seq raw reads using CLC Genomics Workbench (version 21.99)

| **Parameter** | |
| --- | --- |
| Mismatches | 2 |
| Insertion cost | 3 |
| Deletion cost | 3 |
| Length fraction | 0.8 |
| Similarity fraction | 0.8 |
| Maximum number of hits for a read | 10 |
| Strands specificity | Both |

**Supplementary Table 3** Quality trimming summary for *Gardnerella vaginalis*, *Fannyhessea vaginae*, *Prevotella bivia*, and triple-species sequences. Each condition was analyzed in triplicate

**Supplementary Table 4** Genes selected for validation of RNA-sequencing data

| **Genes** | **Description** | **Fold change** |
| --- | --- | --- |
| ***G. vaginalis*** | | |
| EL180_RS06735 | Prevent-host-death protein | 815.08 |
| EL180_RS07255 | Hypothetical protein | 302.58 |
| EL180_RS06515 | Amino acid ABC transporter ATP-binding protein | 162.75 |
| EL180_RS05445 | ATP-binding protein | 67.43 |
| EL180_RS05315 | Type II toxin-antitoxin system RelB/DinJ family antitoxin | 58.12 |
| EL180_RS03670 | ABC transporter ATP-binding protein | 27.09 |
| EL180_RS00450 | InlB B-repeat-containing protein | 4.33 |
| *pdxS* | Pyridoxal 5'-phosphate synthase lyase subunit PdxS | 2.67 |
| EL180_RS06455 | Glycosyltransferase | -2.36 |
| EL180_RS02315 | FIVAR domain-containing protein | -2.41 |
| EL180_RS03180 | Glutamine amidotransferase | -2.49 |
| *glnA*_1 | Type I glutamate--ammonia ligase | -2.60 |
| *vly* | Cholesterol-dependent cytolysin vaginolysin | -2.70 |
| EL180_RS06075 | ABC transporter substrate-binding protein | -3.25 |
| EL180_RS04605 | MFS transporter | -3.41 |
| EL180_RS03360 | CrcB family protein | -14.14 |
| ***F. vaginae*** | | |
| I6G91_01720 | Metal ABC transporter permease | 3.04 |
| I6G91_02215 | DMT family transporter | -2.45 |
| I6G91_03670 | MATE family efflux transporter | -2.62 |
| I6G91_01665 | VanZ family protein | -3.19 |
| ***P. bivia*** | | |
| *pdxT* | Pyridoxal 5'-phosphate synthase glutaminase subunit PdxT | 3.43 |
| PREBIDRAFT_RS04130 | Hypothetical protein | 2.16 |
| PREBIDRAFT_RS02100 | SGNH/GDSL hydrolase family protein | 2.02 |

**Supplementary Table 5** List of primers designed for the confirmation of RNA-seq data by qPCR

| **Genes** | **Primers** | **Efficiency at 60°C** | **Melting temperature** | **Priming strategy for cDNA synthesis** |
| --- | --- | --- | --- | --- |
| ***G. vaginalis*** | | | |  |
| EL180_RS06735 | FW: TGTCGATAAATACGGTTCAGCTG | 93 | 80-80.5 | GSP |
|  | RV: TCCGATAAAGCCATGACCTTATC |  |  |  |
| EL180_RS07255 | FW: CTTTGTGTCGCGCGATATTCTT | 100 | 79-79.5 | GSP |
|  | RV: AGCCCTGGTACTTTAAGAACATCA |  |  |  |
| EL180_RS06515^a^ | FW: TCGTCATATGGAGCAGCAAGAA | 100 | 83.5-84 | GSP |
|  | RV: CCTTCTTTTGCAAGCTCAACCA |  |  |  |
| EL180_RS05445^a^ | FW: CAGTAGGCTTCTCAAACGTTGC | 98 | 78.5-79 | GSP |
|  | RV: GAGTCTCTTCGCTAGCCATCAA |  |  |  |
| EL180_RS05315 | FW: ACTATTCCAAAGTCACTTCCGGCAC | 100 | 80-80.5 | Random |
|  | RV: AACTATCGCCACAAACCGCCTG |  |  |  |
| EL180_RS03670 | FW: AAGTCGTTGCCAGATCTTTACG | 99 | 79-79.5 | Random |
|  | RV: TGTCGGAAGCAAAACCAGATTG |  |  |  |
| EL180_RS00450 | FW: AAACAGCACAGTTCCAAGCCCG | 96 | 80-80.5 | Random |
|  | RV: TGCCACAGACTCATCCTTCGCA |  |  |  |
| *pdxS* | FW: TGCTTCCATGATTCGCACAAAG | 99 | 84-84.5 | Random |
|  | RV: TTTACAAGATCGAAAGGCACGC |  |  |  |
| EL180_RS06455 | FW: GTGGTTCTGGGTTATGGACGAT | 98 | 81.5 - 82 | Random |
|  | RV: CGCTTAAACAATCCGCCTTCAA |  |  |  |
| EL180_RS02315 | FW: AAAGCTCTTGATGGTGACGCTA | 98 | 81.5-82 | Random |
|  | RV: GCAGCATTGTTCCTGGTTTCAT |  |  |  |
| EL180_RS03180 | FW: TGATGGCGAAAATTGCGGAAAT | 100 | 82.5 | Random |
|  | RV: TTGGTGCAAATTCTCCGTAACG |  |  |  |
| *glnA*_1 | FW: ATACATTCCGCGCTCAAGAGAA | 100 | 81-81.5 | Random |
|  | RV: AGCATGCTTGAACAGATGGGTA |  |  |  |
| *vly* | FW: ACCAAGAGCTCTGTAACTTCCG | 96 | 83.5 | Random |
|  | RV: ATCTTCAGCGGAACACCAATCT |  |  |  |
| EL180_RS06075 | FW: GCTGTAGATGCGCTTTCTTCTG | 100 | 82-82.5 | Random |
|  | RV: TCCGCTCACCATTCATGTTCAT |  |  |  |
| EL180_RS04605 | FW: CGTTCTTCTTTACCGCGGAATC | 95 | 82-82.5 | GSP |
|  | RV: AGCTCCAACTAAAGATCCGCAT |  |  |  |
| EL180_RS03360^a^ | FW: GGGCGCGTTTATAACCATGATT | 100 | 79-79.5 | GSP |
|  | RV: TGAAAACGTTGAAAATCCGCCT |  |  |  |
| ***F. vaginae*** | | | |  |
| I6G91_01720 | FW: CGTGGGTCTTTGTTTTGGGTTT | 100 | 83-83.5 | Random |
|  | RV: ATGCCAGCTAAACCGATTGAAG |  |  |  |
| I6G91_02215 | FW: GTAGGCTTACAAACCGCACAAG | 96 | 83 | Random |
|  | RV: AAAGGCAGCACAATTAAAGCGT |  |  |  |
| I6G91_03670 | FW: TATGGCAGTGTTTCCCGATGAA | 99 | 83 | Random |
|  | RV: GGCAACAACGTGACCATACAAA |  |  |  |
| I6G91_01665 | FW: AGTGCCCATATCCTTGAGTATACGGT | 96 | 80-80.5 | Random |
|  | RV: GGCCACTTCTACCAGGAACAAACA |  |  |  |
| ***P. bivia*** | | | |  |
| *pdxT* | FW: ACAGCGGCTAGGGATCGACTTC | 93 | 84-84.5 | Random |
|  | RV: GCACAAGTACCAAAGACGGGCA |  |  |  |
| PREBIDRAFT_RS04130 | FW: GGTAGCCTCTCTCGTTTACACC | 100 | 82.5-83 | Random |
|  | RV: ATTGGCACTTACGGAGACTTGT |  |  |  |
| PREBIDRAFT_RS02100 | FW: CAGACACAAACCTACGAGCAGA | 100 | 81.5 | Random |
|  | RV: GCCAAAGCCCAATCACGAATAG |  |  |  |

GSP: Gene-specific primers.

^a^The primers indicated different amplified products on the triple-species biofilms, as described in Supplementary Table 6.

**Supplementary Table 6** Comparison of the results obtained by RNA-seq RPKM and qPCR (normalization to the 16S gene) using the same RNA utilized for RNA-seq (technical validation)

| **RNA-seq vs qPCR** | | | | | | | | | | | | |
| --- | --- | --- | --- | --- | --- | --- | --- | --- | --- | --- | --- | --- |
| ***G. vaginalis*** | **Gv1** | | **Gv2** | | **Gv3** | | **Mix1** | | **Mix2** | | **Mix3** | |
|  | **RPKM** | **E^-ΔCT^** | **RPKM** | **E^-ΔCT^** | **RPKM** | **E^-ΔCT^** | **RPKM** | **E^-ΔCT^** | **RPKM** | **E^-ΔCT^** | **RPKM** | **E^-ΔCT^** |
| EL180_RS06735 | 3.65 | 1.26×10^-3^ | 5.98 | 6.77×10^-5^ | 6.32 | 7.49×10^-4^ | 7314.83 | 1.41×10^-1^ | 2880.40 | 4.08 ×10^-2^ | 2806.68 | 2.88×10^-2^ |
| EL180_RS07255 | 1.53 | 4.88×10^-4^ | 4.01 | 3.82×10^-5^ | 3.84 | 2.77×10^-4^ | 1159.14 | 8.20×10^-3^ | 899.79 | 4.97×10^-3^ | 777.86 | 3.82×10^-3^ |
| EL180_RS06515 | 5.08 | 8.01×10^-5^ | 6.88 | 2.58×10^-5^ | 8.24 | 7.66×10^-5^ | 1717.88 | ^a^ | 839.87 | ^a^ | 728.59 | ^a^ |
| EL180_RS05445 | 2.33 | 2.38×10^-4^ | 4.87 | 7.66×10^-6^ | 4.03 | 1.12×10^-4^ | 425.72 | ^a^ | 173.96 | ^a^ | 157.09 | ^a^ |
| EL180_RS05315 | 33.96 | 3.68×10^-5^ | 94.45 | 1.15×10^-5^ | 94.73 | 2.41×10^-5^ | 6888.52 | 6.71×10^-4^ | 3121.14 | 1.85×10^-4^ | 2958.25 | 2.80×10^4^ |
| EL180_RS03670 | 3.58 | 1.49×10^-6^ | 5.22 | 1.66×10^-7^ | 4.28 | 1.02×10^-6^ | 158.42 | ND | 96.44 | 1.74×10^-5^ | 99.41 | 1.81×10^-5^ |
| EL180_RS00450 | 49.71 | 3.41×10^-6^ | 36.68 | 6.27×10^-6^ | 52.71 | 4.65×10^-6^ | 287.66 | 6.65×10^-6^ | 170.76 | 8.17×10^-6^ | 143.94 | 5.44×10^-6^ |
| *pdxS* | 41.12 | 1.93×10^-6^ | 75.97 | 2.79×10^-6^ | 65.47 | 2.91×10^-6^ | 196.62 | 6.44×10^-6^ | 146.39 | 1.46×10^-5^ | 143.82. | 2.08×10^-5^ |
| EL180_RS06455 | 17.79 | 1.78×10^-6^ | 29.46 | 1.50×10^-6^ | 18.69 | 1.47×10^-6^ | 5.92 | ND | 12.32 | 9.77×10^-5^ | 9.65 | 8.54×10^-6^ |
| EL180_RS02315 | 526.32 | 5.54×10^-5^ | 827.62 | 1.59×10^-4^ | 730.97 | 5.37×10^-5^ | 140.69 | ^a^ | 370.37 | 6.41×10^-5^ | 355.25 | 9.67×10^-5^ |
| EL180_RS03180 | 17.43 | 5.95×10^-6^ | 27.36 | 4.36×10^-6^ | 19.85 | 5.85×10^-6^ | 11.12 | ND | 4.75 | 3.99×10^-5^ | 10.08 | 4.79×10^-5^ |
| *glnA*_1 | 21.08 | 1.53×10^-6^ | 36.10 | 1.30×10^-6^ | 31.88 | 1.71×10^-6^ | 3.35 | ND | 15.40 | 1.95×10^-5^ | 15.50 | 2.02×10^-5^ |
| *vly* | 188.51 | 2.53×10^-5^ | 270.08 | 6.37×10^-5^ | 182.18 | 2.63×10^-5^ | 42.45 | 3.49×10^-5^ | 96.69 | 3.96×10^-5^ | 98.07 | 3.90×10^-5^ |
| EL180_RS06075 | 7.04 | 3.94×10^-6^ | 12.34 | 9.16×10^-7^ | 9.04 | 2.83×10^-6^ | 3.56 | 7.16×10^-6^ | 2.28 | 1.81×10^-5^ | 2.90 | 3.70×10^-5^ |
| EL180_RS04605 | 21.67 | 5.80×10^-4^ | 32.21 | 1.43×10^-4^ | 18.19 | 3.61×10^-4^ | 3.25 | 8.82 ×10^-5^ | 9.03 | 5.79×10^-4^ | 8.84 | 4.09×10^-4^ |
| EL180_RS03360 | 2.10 | 6.75×10^-4^ | 7.09 | 2.96×10^-5^ | 6.04 | 2.75×10^-4^ | 0.00 | ^a^ | 1.14 | ^a^ | 0.00 | ^a^ |
| ***F. vaginae*** | **Fv1** | | **Fv2** | | **Fv3** | | **Mix1** | | **Mix2** | | **Mix3** | |
|  | **RPKM** | **E^-ΔCT^** | **RPKM** | **E^-ΔCT^** | **RPKM** | **E^-ΔCT^** | **RPKM** | **E^-ΔCT^** | **RPKM** | **E^-ΔCT^** | **RPKM** | **E^-ΔCT^** |
| I6G91_01720 | 100.63 | 4.50×10^-5^ | 88.55 | 6.23×10^-5^ | 119.37 | 6.70×10^-5^ | 315.68 | 2.38×10^-4^ | 326.36 | 2.55×10^-4^ | 295.83 | 2.34×10^-4^ |
| I6G91_02215 | 39.39 | 1.06×10^-5^ | 39.53 | 1.73×10^-5^ | 57.21 | 2.46×10^-5^ | 12.38 | 5.10×10^-6^ | 21.52 | 7.75×10^-6^ | 21.59 | 1.21×10^-5^ |
| I6G91_03670 | 23.94 | 1.51×10^-6^ | 21.07 | 4.92×10^-6^ | 32.75 | 3.89×10^-6^ | 7.35 | 1.91×10^-6^ | 11.28 | 5.27×10^-6^ | 10.99 | 3.03×10^-6^ |
| I6G91_01665 | 56.35 | 1.02×10^-5^ | 63.71 | 1.39×10^-5^ | 100.94 | 2.33×10^-5^ | 22.36 | 3.72×10^-6^ | 23.76 | 4.97×10^-6^ | 23.14 | 4.40×10^-6^ |
| ***P. bivia*** | **Pb1** | | **Pb2** | | **Pb3** | | **Mix1** | | **Mix2** | | **Mix3** | |
|  | **RPKM** | **E^-ΔCT^** | **RPKM** | **E^-ΔCT^** | **RPKM** | **E^-ΔCT^** | **RPKM** | **E^-ΔCT^** | **RPKM** | **E^-ΔCT^** | **RPKM** | **E^-ΔCT^** |
| *pdxT* | 317.75 | 9.21×10^-5^ | 316.69 | 1.04×10^-4^ | 348.96 | 8.22×10^-5^ | 589.12 | 1.33×10^-4^ | 1496.00 | 2.99×10^-4^ | 1285.54 | 2.74×10^-4^ |
| PREBIDRAFT_RS04130 | 69.57 | 1.79×10^-5^ | 49.62 | 2.95×10^-5^ | 46.21 | 7.26×10^-6^ | 121.38 | 3.29×10^-5^ | 131.50 | 1.06×10^-5^ | 104.38 | 1.76×10^-5^ |
| PREBIDRAFT_RS02100 | 31.84 | 1.36×10^-5^ | 42.97 | 1.01×10^-5^ | 41.05 | 1.03×10^-6^ | 76.98 | ND | 76.33 | 3.52×10^-6^ | 80.90 | 6.22×10^-6^ |

ND: non-detected (cycle threshold values above 38).

^a^For the samples indicated, the quantification could not be determined because the primers amplified different products or products with different melting temperatures.


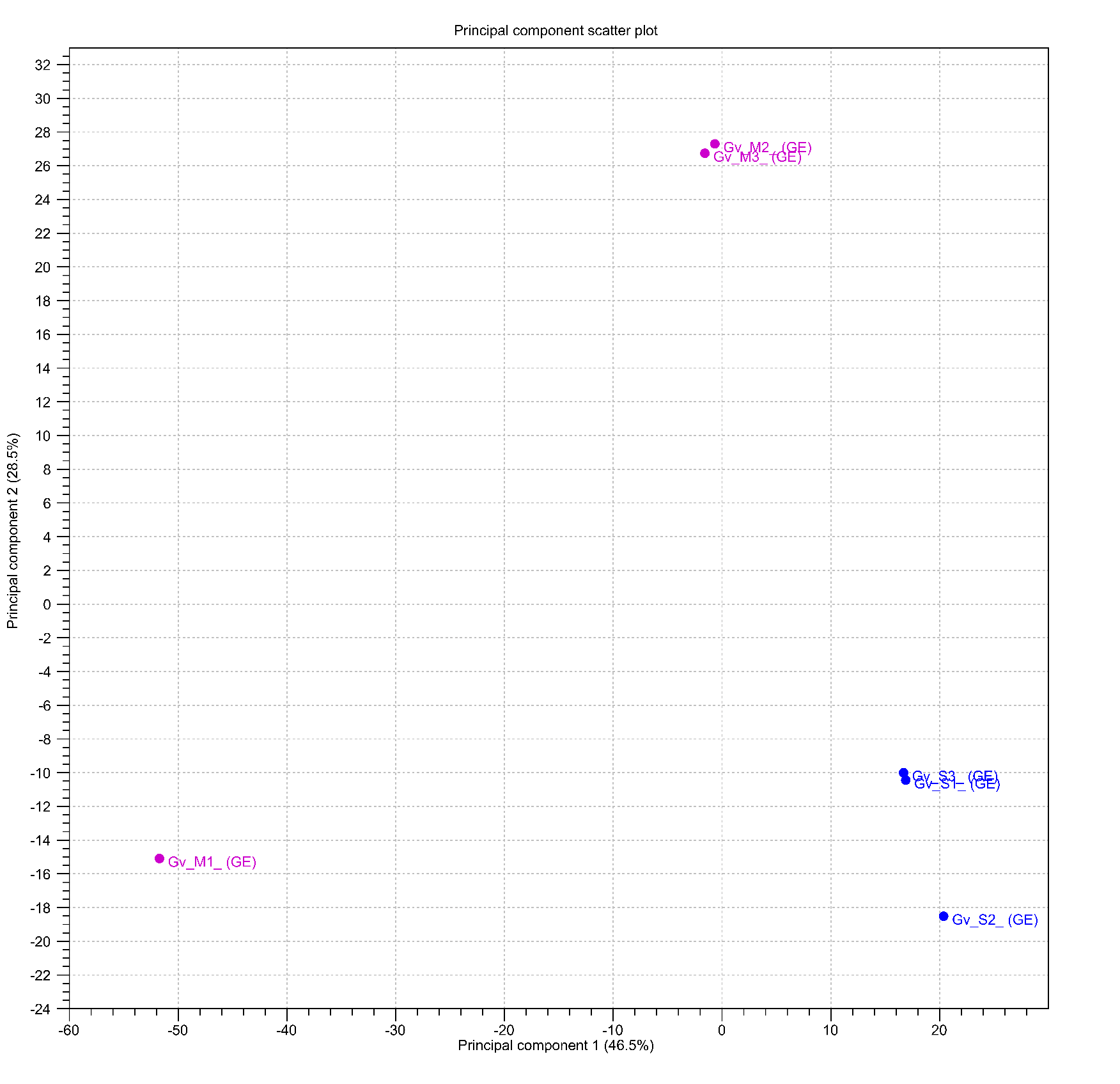
**Supplementary Fig. 1** Principal component scatter plot for *Gardnerella vaginalis*. The blue points represent the triplicates of single-species biofilms (S1, S2, S3) and the pink points represent the triplicates of triple-species biofilms (M1, M2, M3). Figure plotted using the CLC genomics software.

**
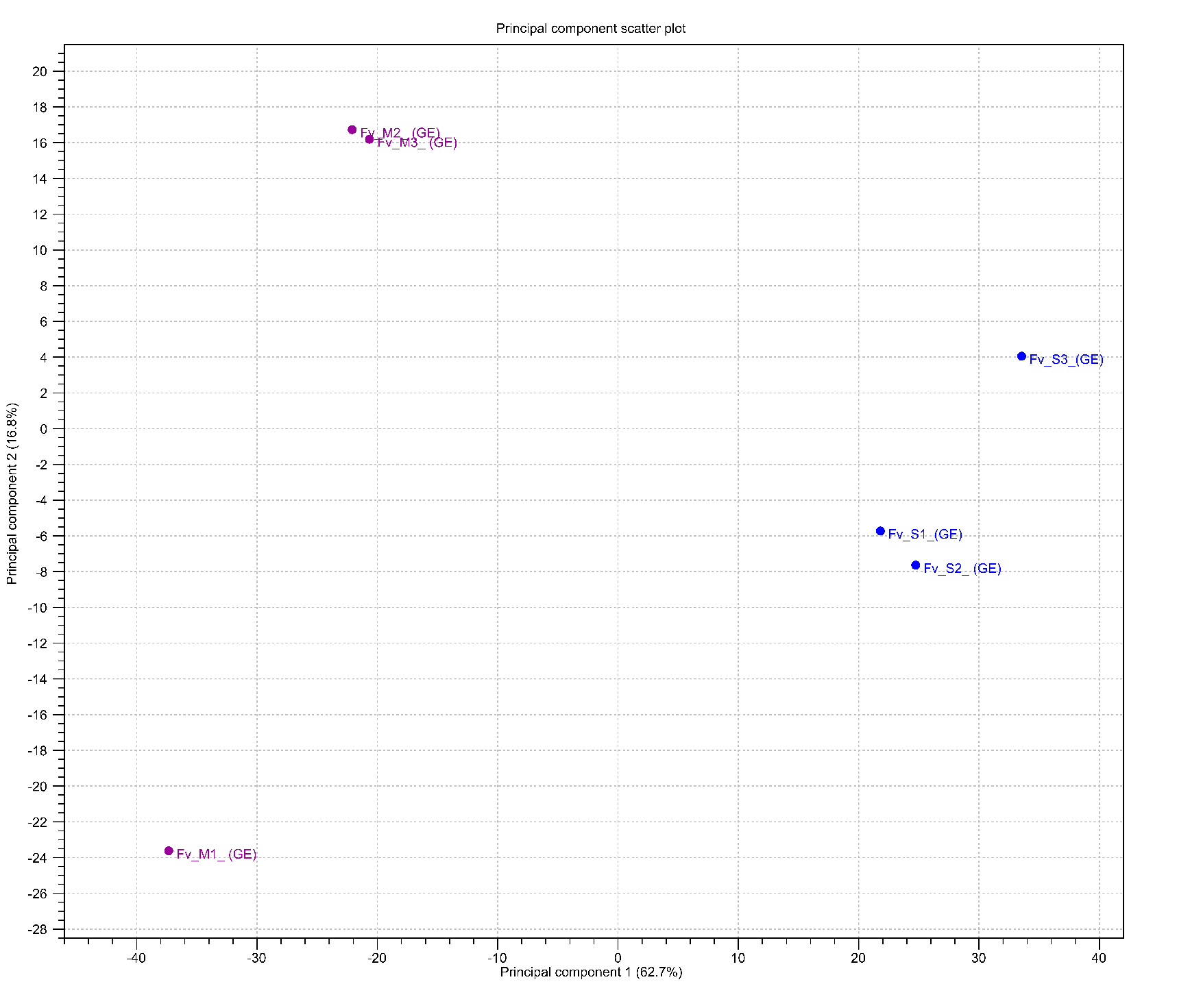
Supplementary Fig. 2** Principal component scatter plot for *Fannyhessea vaginae*. The blue points represent the triplicates of single-species biofilms (S1, S2, S3) and the pink points represent the triplicates of triple-species biofilms (M1, M2, M3). Figure plotted using the CLC genomics software.


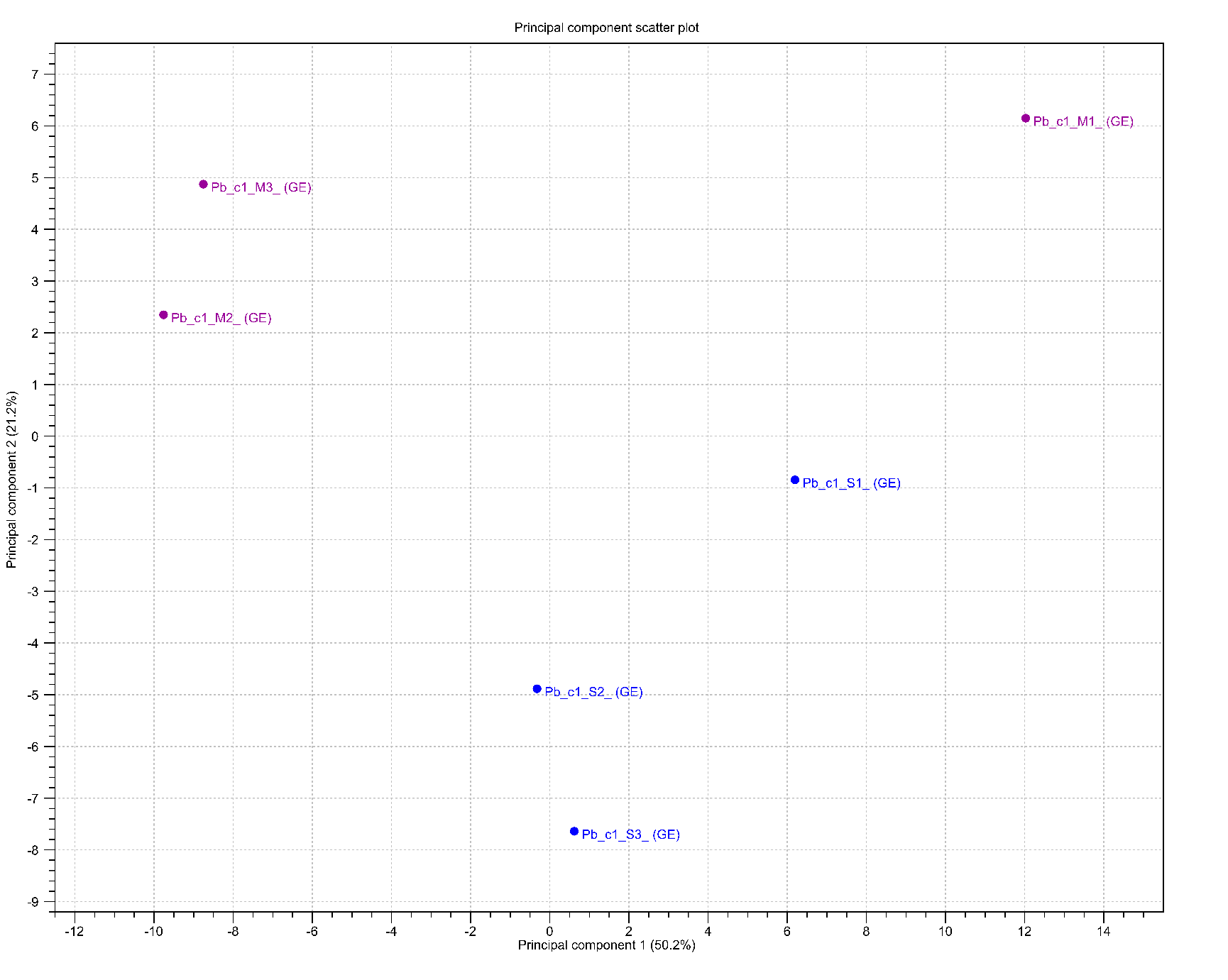
**Supplementary Fig. 3** Principal component scatter plot for scaffold 1 of *Prevotella bivia*. The blue points represent the triplicates of single-species biofilms (S1, S2, S3) and the pink points represent the triplicates of triple-species biofilms (M1, M2, M3). Figure plotted using the CLC genomics software.


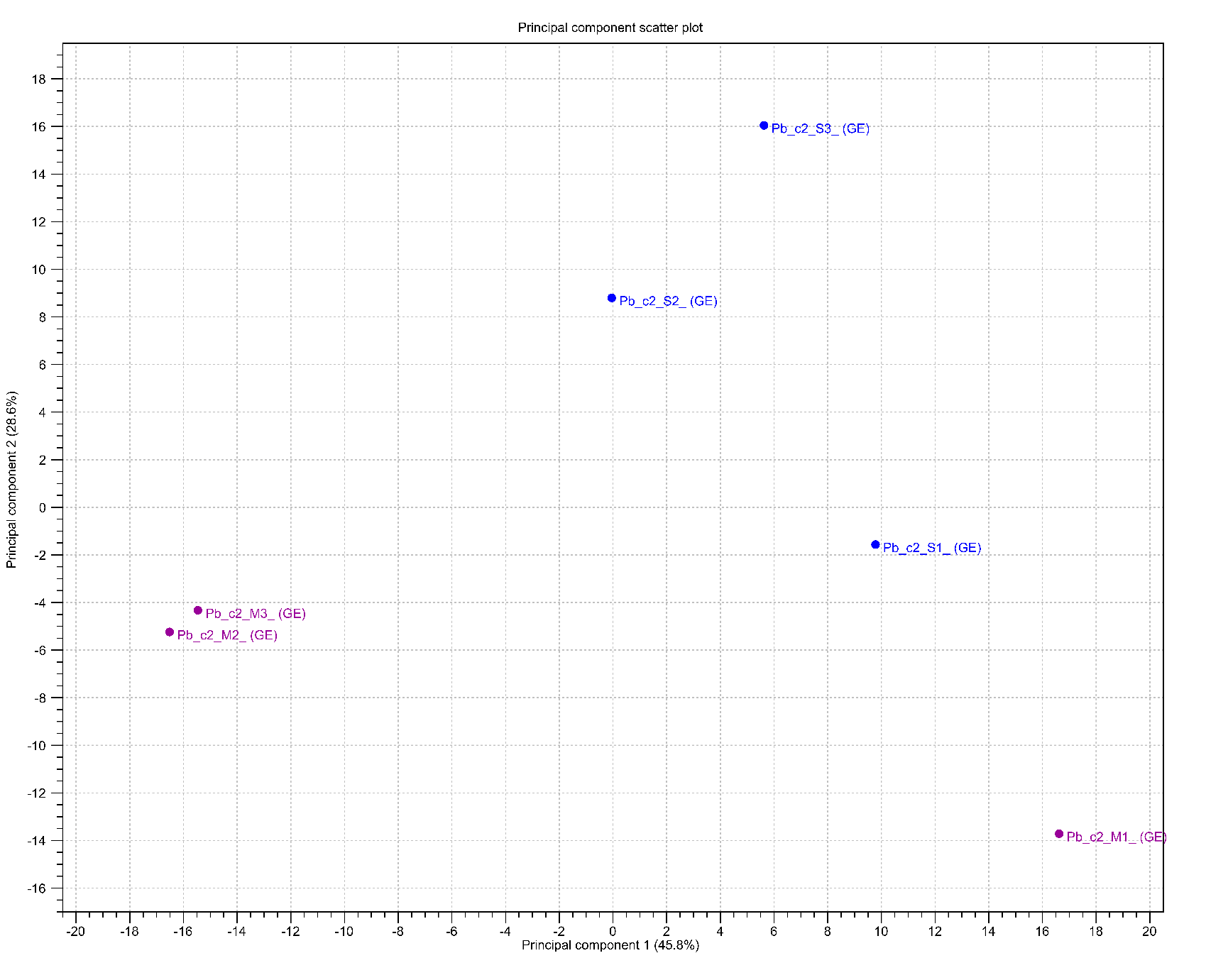
**Supplementary Fig. 4** Principal component scatter plot for scaffold 2 of *Prevotella bivia*. The blue points represent the triplicates of single-species biofilms (S1, S2, S3) and the pink points represent the triplicates of triple-species biofilms (M1, M2, M3). Figure plotted using the CLC genomics software.


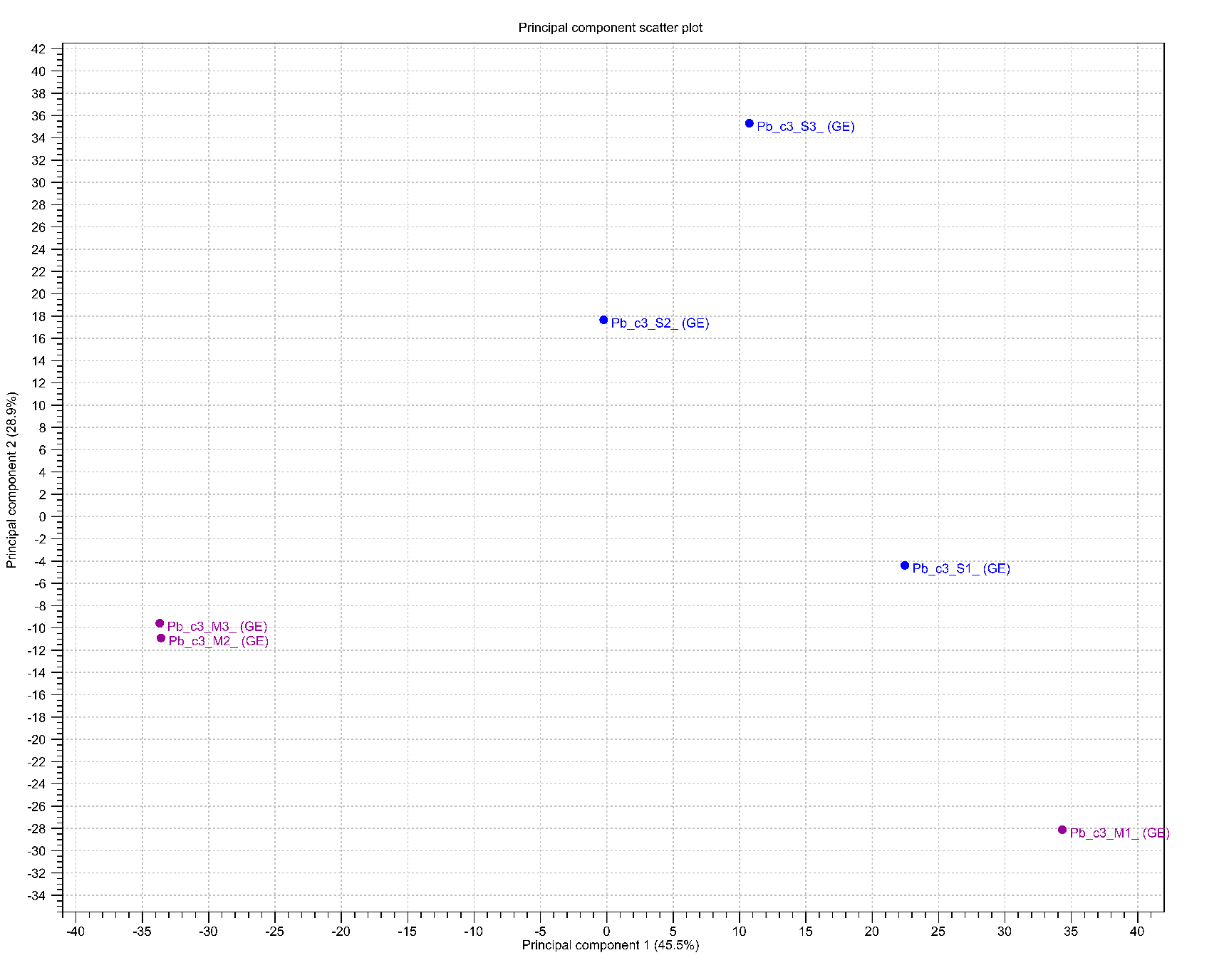
**Supplementary Fig. 5** Principal component scatter plot for scaffold 3 of *Prevotella bivia*. The blue points represent the triplicates of single-species biofilms (S1, S2, S3) and the pink points represent the triplicates of triple-species biofilms (M1, M2, M3). Figure plotted using the CLC genomics software.

**
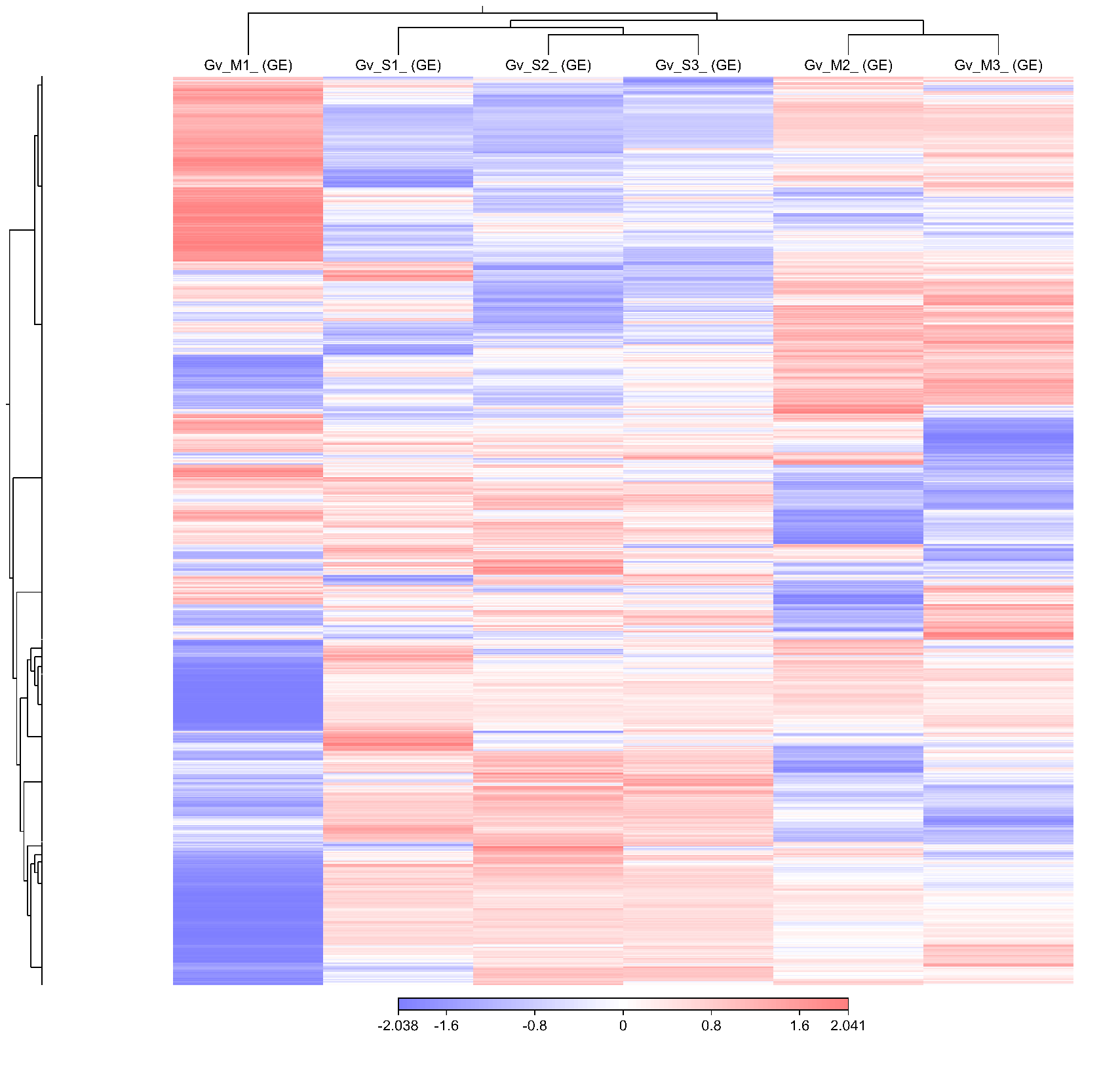
Supplementary Fig. 6** Heatmap of differentially expressed genes in *Gardnerella vaginalis*. Triplicates of single-species biofilms are represented by S1, S2, and S3 and the triplicates of triple-species biofilms are represented by M1, M2 and M3. The color scale indicates the gene expression values from most downregulated (blue) to most upregulated (red). Figure plotted using the CLC genomics software.


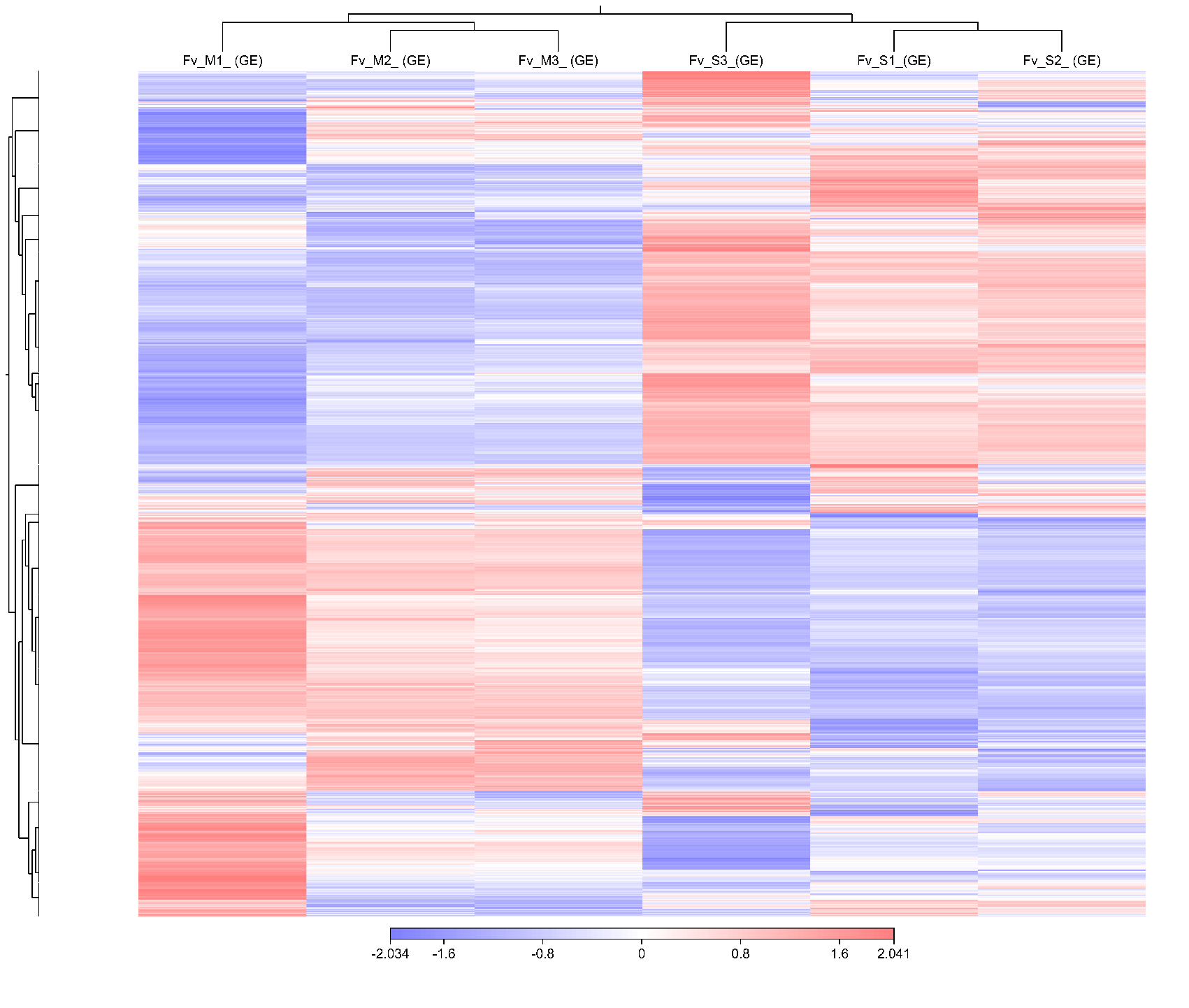
**Supplementary Fig. 7** Heatmap of differentially expressed genes in *Fannyhessea vaginae*. Triplicates of single-species biofilms are represented by S1, S2, and S3 and the triplicates of triple-species biofilms are represented by M1, M2 and M3. The color scale indicates the gene expression values from most downregulated (blue) to most upregulated (red). Figure plotted using the CLC genomics software.


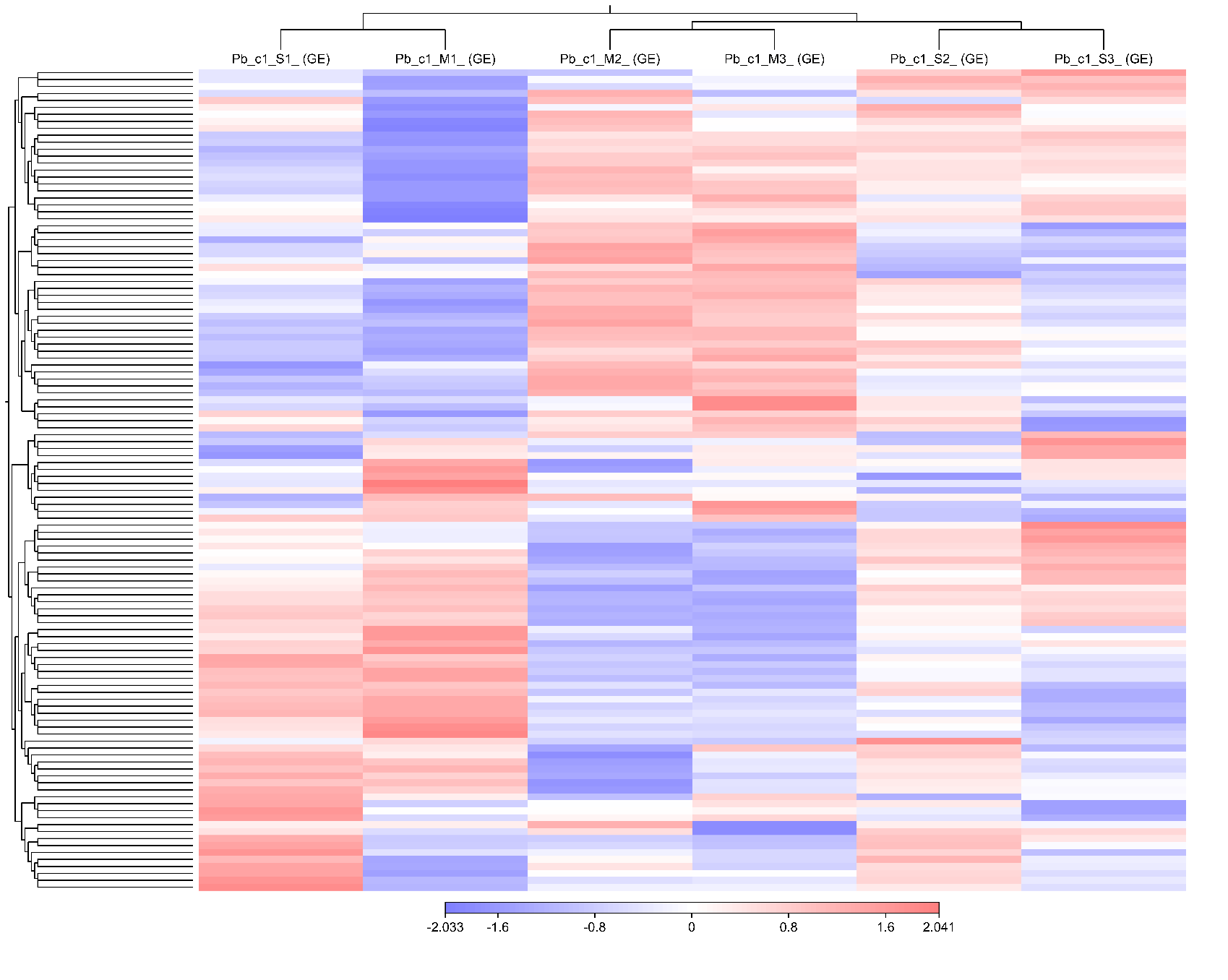
**Supplementary Fig. 8** Heatmap of differentially expressed genes on scaffold 1 of *Prevotella bivia*. Triplicates of single-species biofilms are represented by S1, S2, and S3 and the triplicates of triple-species biofilms are represented by M1, M2 and M3. The color scale indicates the gene expression values from most downregulated (blue) to most upregulated (red). Figure plotted using the CLC genomics software.


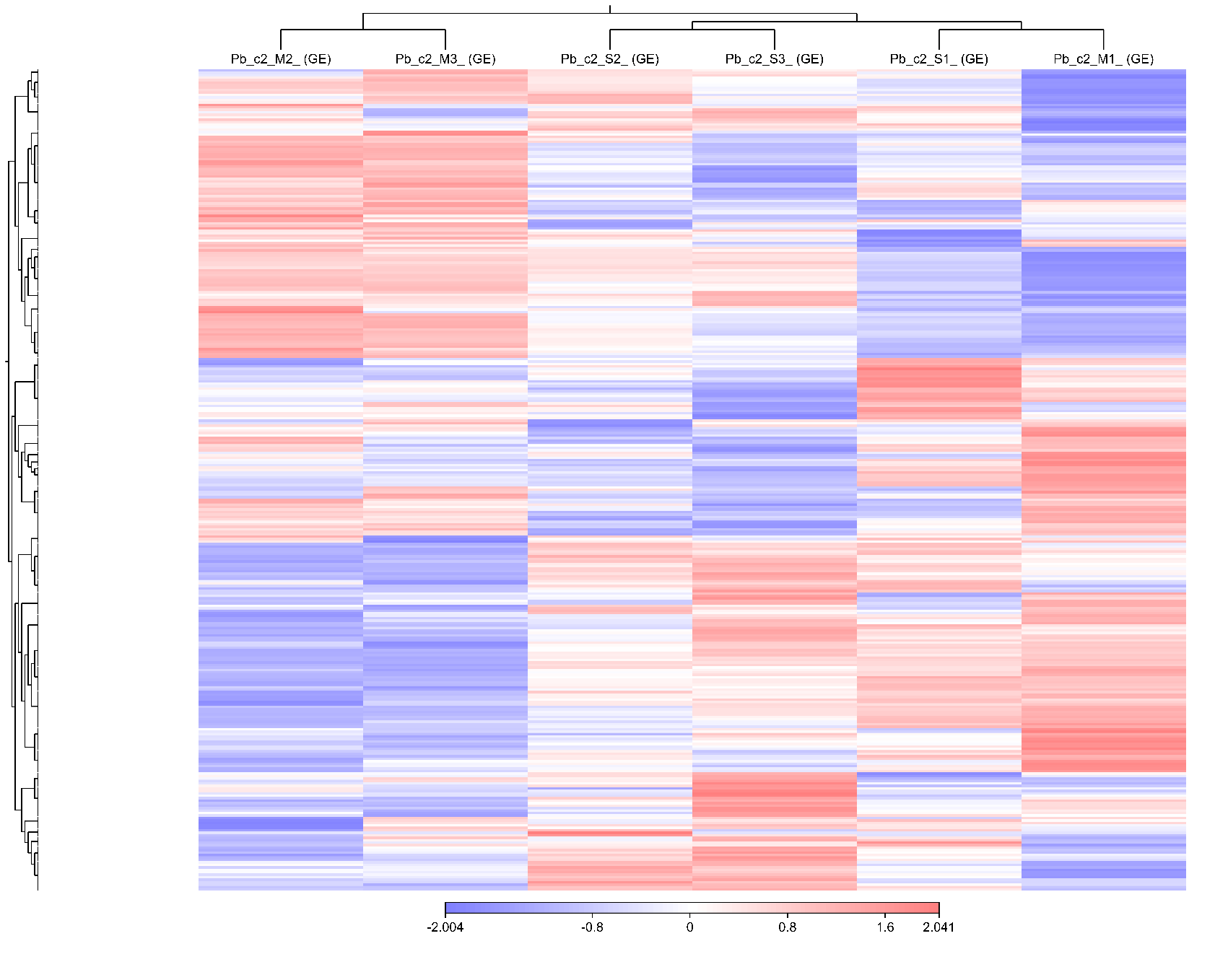
**Supplementary Fig. 9** Heatmap of differentially expressed genes on scaffold 2 of *Prevotella bivia*. Triplicates of single-species biofilms are represented by S1, S2, and S3 and the triplicates of triple-species biofilms are represented by M1, M2 and M3. The color scale indicates the gene expression values from most downregulated (blue) to most upregulated (red). Figure plotted using the CLC genomics software.


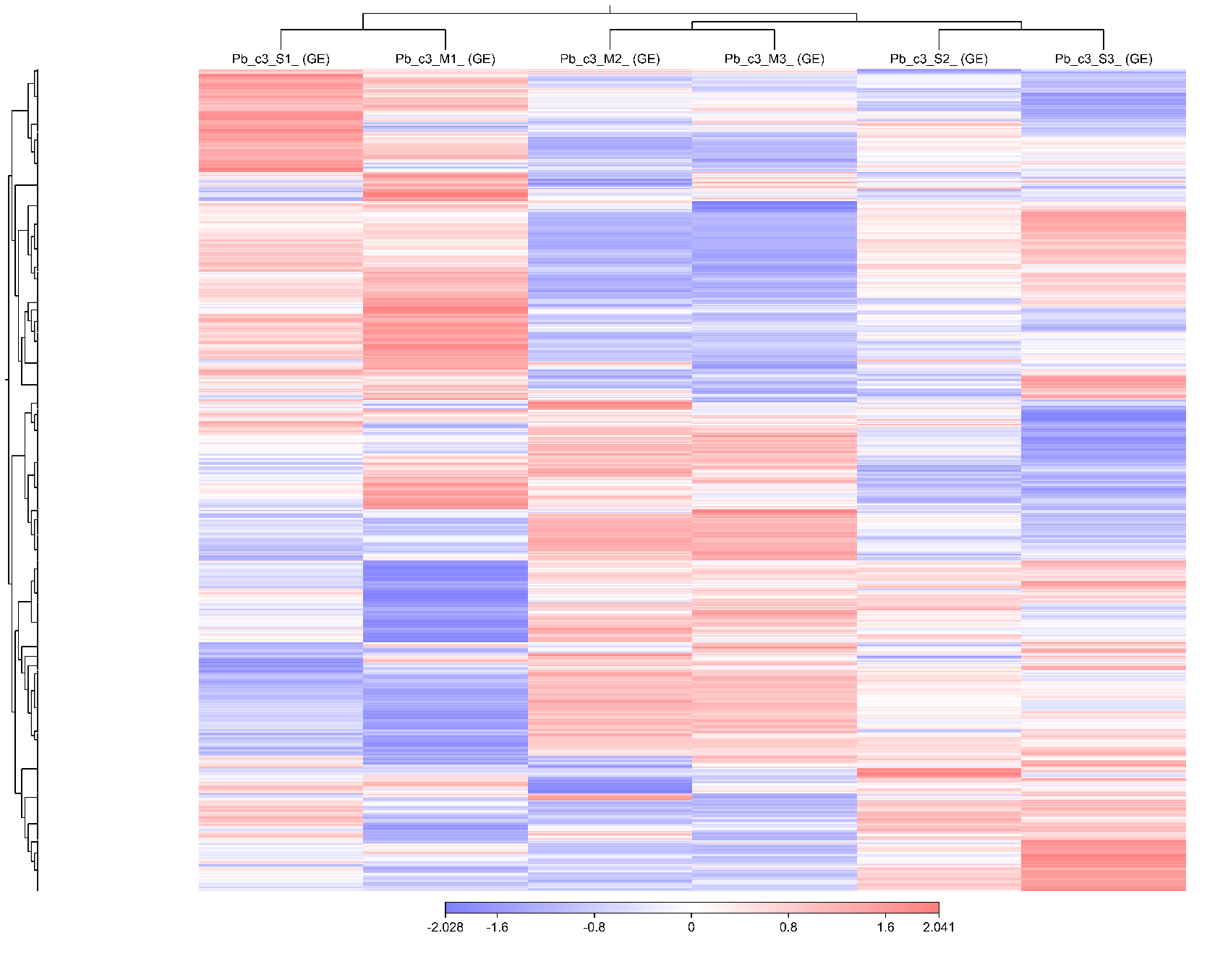
**Supplementary Fig. 10** Heatmap of differentially expressed genes on scaffold 3 of *Prevotella bivia*. Triplicates of single-species biofilms are represented by S1, S2, and S3 and the triplicates of triple-species biofilms are represented by M1, M2 and M3. The color scale indicates the gene expression values from most downregulated (blue) to most upregulated (red). Figure plotted using the CLC genomics software.


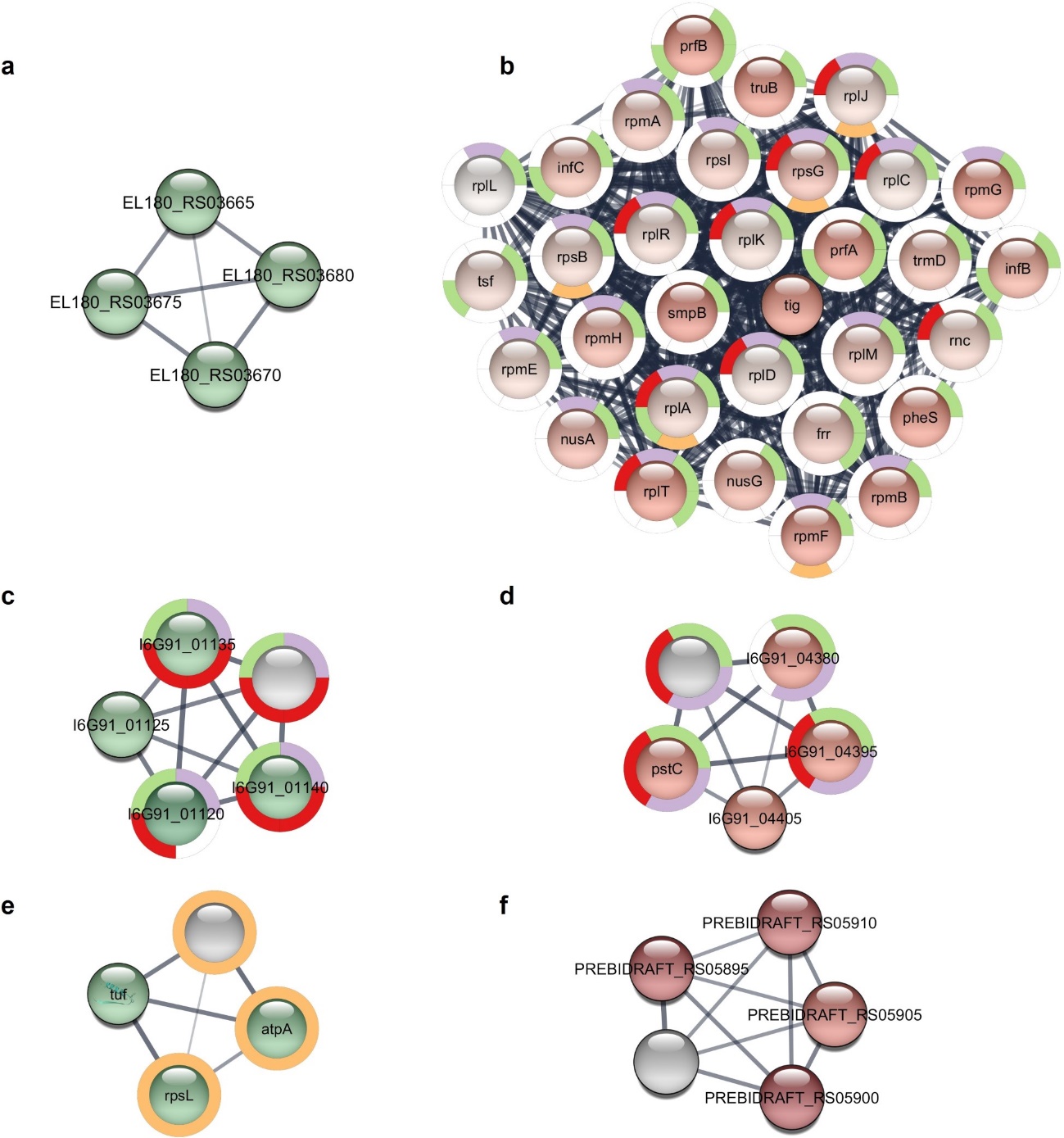


**Supplementary Fig. 11** Main clusters in upregulated and downregulated genes in *Gardnerella vaginalis*, *Fannyhessea vaginae*, and *Prevotella bivia*. The clusters were generated using the MCODE app in Cytoscape and the enrichment results were obtained with the enrichment function from STRING app. Upregulated and downregulated genes are represented by green and red nodes, respectively, for *G. vaginalis* (a, b), *F. vaginae* (c, d), and *P. bivia* (e, f). Results of enrichment are represented by different colors around the nodes where green represents biological processes, orange represents cellular components, red represents molecular functions, and lilac represents KEGG pathways.
